# Identifying General Tumor and Specific Lung Cancer Biomarkers by Transcriptomic Analysis

**DOI:** 10.3390/biology11071082

**Published:** 2022-07-20

**Authors:** Beatriz Andrea Otálora-Otálora, Daniel Alejandro Osuna-Garzón, Michael Steven Carvajal-Parra, Alejandra Cañas, Martín Montecino, Liliana López-Kleine, Adriana Rojas

**Affiliations:** 1 Facultad de Medicina, Universidad Nacional de Colombia, Bogotá 11001, Colombia; baotalorao@unal.edu.co; 2 Departamento de Estadística, Universidad Nacional de Colombia, Bogotá 11001, Colombia; daosunag@unal.edu.co (D.A.O.-G.); mscarvajalp@unal.edu.co (M.S.C.-P.); 3 Departamento de Medicina Interna, Facultad de Medicina, Pontificia Universidad Javeriana, Bogotá 110211, Colombia; alejandra.canas@javeriana.edu.co; 4 Unidad de Neumología, Hospital Universitario San Ignacio, Bogotá 110211, Colombia; 5 Institute of Biomedical Sciences, Facultad de Medicina y Facultad de Ciencias de la Vida, Universidad Andres Bello, Santiago 8370134, Chile; mmontecino@unab.cl; 6 Instituto de Genética Humana, Facultad de Medicina, Pontificia Universidad Javeriana, Bogotá 110211, Colombia

**Keywords:** lung cancer (LC), breast cancer (BC), leukemia (LK), differentially expressed genes (DEGs), coexpression networks, early detection and prognosis biomarkers

## Abstract

**Simple Summary:**

An adequate bioinformatic pipeline is a valuable tool for understanding cancer mechanisms and identifying transcriptomic biomarkers of cancer and specific to lung cancer. The bioinformatic pipeline was applied to analyze multiple transcriptomic studies and to identify an important group of winning transcription factors coexpressed in gene networks of lung cancer, breast cancer and leukemia, capable of forming coregulatory complexes associated with the regulation of genes involved in tumorigenic processes related to the acquisition of the hallmarks of cancer, as well as lung cancer patients survival. The establishment of a general and specific transcriptional regulatory network is essential to develop key molecular tools for prevention, early diagnosis, and treatment aimed at precision personalized medicine of cancer.

**Abstract:**

The bioinformatic pipeline previously developed in our research laboratory is used to identify potential general and specific deregulated tumor genes and transcription factors related to the establishment and progression of tumoral diseases, now comparing lung cancer with other two types of cancer. Twenty microarray datasets were selected and analyzed separately to identify hub differentiated expressed genes and compared to identify all the deregulated genes and transcription factors in common between the three types of cancer and those unique to lung cancer. The winning DEGs analysis allowed to identify an important number of TFs deregulated in the majority of microarray datasets, which can become key biomarkers of general tumors and specific to lung cancer. A coexpression network was constructed for every dataset with all deregulated genes associated with lung cancer, according to DAVID’s tool enrichment analysis, and transcription factors capable of regulating them, according to oPOSSUM´s tool. Several genes and transcription factors are coexpressed in the networks, suggesting that they could be related to the establishment or progression of the tumoral pathology in any tissue and specifically in the lung. The comparison of the coexpression networks of lung cancer and other types of cancer allowed the identification of common connectivity patterns with deregulated genes and transcription factors correlated to important tumoral processes and signaling pathways that have not been studied yet to experimentally validate their role in lung cancer. The Kaplan–Meier estimator determined the association of thirteen deregulated top winning transcription factors with the survival of lung cancer patients. The coregulatory analysis identified two top winning transcription factors networks related to the regulatory control of gene expression in lung and breast cancer. Our transcriptomic analysis suggests that cancer has an important coregulatory network of transcription factors related to the acquisition of the hallmarks of cancer. Moreover, lung cancer has a group of genes and transcription factors unique to pulmonary tissue that are coexpressed during tumorigenesis and must be studied experimentally to fully understand their role in the pathogenesis within its very complex transcriptomic scenario. Therefore, the downstream bioinformatic analysis developed was able to identify a coregulatory metafirm of cancer in general and specific to lung cancer taking into account the great heterogeneity of the tumoral process at cellular and population levels.

## 1. Introduction

Cancer is the second leading cause of death around the world [1]. In men, cancer most often affects the lung, prostate, and colorectum, while, in women, it affects the lung, breast, and colorectum [1]. In children, the most common types are blood cancer and cancers related to the brain and lymph nodes [1]. Cancer is a disease characterized by the formation of tumors in bone, skin, tissue, organs, and glands due to an increase in cell growth, as well as the invasion of other tissues [2]. Cancer is a highly heterogeneous disease with different histological and biological characteristics defined by genetic, epigenetic, and transcriptomic features, which determine clinical and treatment outcomes [3]. Most lung cancer cases (85%) are related to the histological type known as non-small cell cancer (NSCLC), followed by small cell carcinoma (SCLC) (15%). The three main subtypes of NSCLC are adenocarcinoma, squamous-cell carcinoma, and large-cell carcinoma [4]. The WHO has classified breast cancer in 20 major types and 18 minor subtypes according to the histopathology: carcinoma in situ (15–30%), invasive carcinoma (70–85%) (invasive ductal carcinoma and invasive lobular carcinoma), and mesenchymal tumors (including sarcoma); the grade (well differentiated (low-grade), moderately differentiated (intermediate-grade), and poorly differentiated (high-grade); and stage (primary tumor, regional lymph nodes, and distant metastases) [5]. Breast cancer also has specific molecular characteristics, such as the activation of human epidermal growth factor receptor 2 (HER2), the activation of hormone receptors, such as estrogen and progesterone, and BRCA mutations [6]. Leukemia is a group of blood cancers that usually begin in the bone marrow and have been classified in four major subtypes (acute lymphoblastic leukemia (ALL), acute myeloid leukemia (AML), chronic lymphocytic leukemia (CLL), and chronic myeloid leukemia (CML)) [7].

The high mortality rates of cancer have been associated with the lack of an early diagnosis based on specific biomarkers able to detect the potential to develop a tumorigenic process in the lung and to develop specific treatments against the disease [2]. The search of key cancer biomarkers is essential for early diagnosis and prognosis, classifying sensitive patients, and predicting the treatment response of every patient [8]. Cancer research has identified a significant number of predictive biomarkers related to the response to a certain treatment for NSCLC patients [9,10]. The identification of genetic risk factors that increase the susceptibility to develop lung cancer is important to develop specific treatments [11]. However, there is still an important limitation in this approach because it treats only a few patients with the associated risk factor, and, therefore, the mortality rate has not shown significant reductions [12].

The dysregulation of gene expression in human cells is under study in many laboratories around the world in the context of different studies of diseases. Currently, transcriptomic analysis combined with large sample sizes have the potential to lead to significant advances in understanding the genetic and regulatory architecture of very complex diseases with risks contributed by multiple gene expression dysregulation. The complex interaction between genes and environment (GXE) is deeply involved in multiple aspects of cancer, from determining an individual’s vulnerability to onset to influencing its response to therapeutic intervention [13]. Consequently, at the molecular level, the ability of a cell to communicate with its environment is essential for guaranteeing the proper molecular functions in human cells and to promote important changes in cell growth, proliferation, differentiation, survival, and death through the activation or repression of a group of genes called immediate early genes (IEGs) [14]. The key components of the GXE interaction are the IEGs, which are known as the “gateway to the genomic response” since IEGs are the first to be activated at the transcription level in response to a wide variety of cellular stimuli from extracellular signals. The main IEG products are early regulators of cell growth and differentiation signals, which essentially are transcription factors (TFs), along with other DNA-binding proteins, cytoskeletal proteins, and receptor subunits [14]. The TFs regulate gene expression by binding to cis-acting DNA regulatory elements, including enhancers, insulators, and promoters of genes, in order to activate or inhibit their transcription [15]. The TFs can be classified into 71 families based on the homology of their DNA-binding domains, but three of them contain more than 50% of the total TFs. The C2H2 zinc finger family has over 600 members, the homeobox family over 200 members, and the bHLH family over 80 members [16]. TFs control expression in a specific spatial, temporal, and sequential manner during development and tissues differentiation; therefore, changes in their expression can lead to the master dysregulation of cell integrity and homeostasis, and, in this way, to the development of complex pathologies [17]. TFs are commonly deregulated in the major class of cancer cells, making them very interesting targets for cancer therapy [18]. The first step, then, is to identify the most important TFs as key biomarkers to be able to study their biological function in normal and cancer contexts in order to move forward in the translational cancer research field.

Our research laboratory has proposed a new methodology based on the identification of common and unique deregulated genes and TFs in cancer, along with the creation of coexpression networks with microarray datasets, using “Coexnet” [19], an R library developed by our research group, to study the complexity of lung cancer and the identification of biomarkers associated with biological processes and signaling pathways related to the acquisition of the hallmarks of cancer during the establishment and progression of the disease [20]. Our bioinformatic pipeline seeks to address the complexity of the disease in terms of the intertumoral and intratumoral variability in each individual and/or population of individuals, as well as the large number of deregulated genes that have been identified with genomic studies, conducting a joint transcriptomic analysis of lung cancer with microarray data, identifying the signaling pathways related to specific diagnosis gene groups in coexpression networks associated with tumorigenesis, which could become good targets for the development of lung cancer drug-oriented treatments. We have already used this bioinformatic pipeline to identify transcription factors that could be related to the establishment and progression of lung cancer, comparing gene expression studies of lung cancer and other lung diseases, and creating coexpression networks of deregulated genes and transcription factors, which are related to the acquisition of the hallmarks of cancer [20]. Now, we want to extend our joint transcriptomic analysis to other types of cancer to identify important biomarkers that are coexpressed in tumorigenic processes in the lung and other tissues affected with the disease, which could become important targets for the development of diagnostic and treatment techniques against cancer and lung cancer.

## 2. Materials and Methods

Twenty sets of expression datasets (microarrays) were selected from the public repositories Gene Expression Omnibus (GEO) from NCBI and Array Express from EBI (Table 1). Ten sets compare tumor tissue from patients with lung cancer and normal lung tissue, five sets compare tumor tissue from patients with breast cancer and normal breast tissue, and five sets compare blood tissue of leukemia patients and normal blood tissue of individuals not affected with the disease. The selected datasets passed the same bioinformatic pipeline developed for the analysis of our previous transcriptomic studies [20]. The process started with all microarray data preprocessing (quality verification, filter, and normalization) [21] to select the best microarray datasets for the analysis, and then we conducted the detection of differentially expressed genes (DEGs) [22] with R language and specialized libraries [19].

The list of all deregulated genes in lung cancer (LC), breast cancer (BC), and leukemia were compared to identify those varying in the same sense (upregulated or downregulated in the pathology with respect to healthy tissue) in the majority of microarray datasets (at least eight of the ten sets of lung cancer and in at least one of the other types of cancer) to establish our “Common winning DEGs” and in the majority of LC microarray datasets (at least seven of the ten sets of lung cancer and none of the other types of cancer) to establish our “Unique LC winning DEGs”. These were highlighted in the functional and enrichment analysis, classifying those involved in tumorigenic processes with the online DAVID tool (Bioinformatics Resources 6.8, NIAID/NIH” (https://david.ncifcrf.gov/summary.jsp) (accessed on 15 June 2019) [44] and a corrected *p* value ≤ 0.05 with Benjamini’s method [45], along with a very extensive literature search of all the common and unique deregulated winner DEGs related to tumoral processes, in order to validate its association with the acquisition of the hallmarks of cancer.

The coexpression networks of common winning DEGs of cancer (LC&OC and unique lung-cancer-related winning DEGs (LCII) and all transcription factors capable to regulate them according to oPOSSUM database (https://github.com/wassermanlab/oPOSSUM3) (accessed on 14 July 2019) were conducted under the same parameters for the construction of the similarity matrix and the clustering coefficient developed by our research group [21] and used in our previous study with GSE19804 microarray, the lung cancer dataset with the best number of controls and cases. Gedevo [46] and the R library “Coexnet’’ were used to compare the coexpression networks of all datasets to identify all common connectivity patterns (CCPs) between them [19]. Cytoscape [47] and iRegulon [48] applications allowed the analysis and identification of TFs for every CCP.

The coregulatory networks of winning TFs were completed with R library “Coregnet’’ [49] in a selected dataset of lung cancer (GSE19804), breast cancer (GSE3744), and leukemia (GSE6691), where all the winning TFs were deregulated. The Kaplan–Meier estimator (http://kmplot.com/analysis/index.php?p=service&cancer=lung) (accessed on 23 June 2022) was used to measure the overall survival (OS) of lung cancer patients according to the deregulation of the top winning TFs [50].

## 3. Results

### 3.1. Differentially Expressed Genes (DEGs) Common between the 3 Types of Cancer, and Unique Lung Cancer DEGs

**Figure 1:**
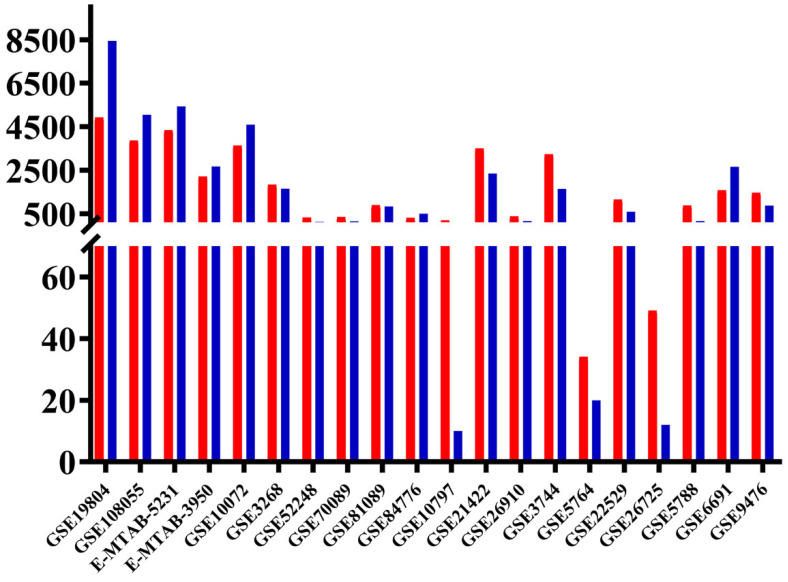
Number of differentially expressed genes (DEGs) in lung cancer LC and other types of cancer (OTC) microarray studies. Overregulated in blue and downregulated in red. The image was made using GraphPad Prism version 8.00 for Windows, GraphPad Software, La Jolla, California, USA, www.graphpad.com (accessed on 12 June 2019).

In general, there are more DEGs in lung cancer than in other types of cancer, and there is great variability in overregulated and downregulated genes in every type of cancer (Figure 1) (Supplementary Table S1). The identification of 296 genes that were equally differentially expressed in lung cancer and other lung diseases, and 98 genes equally deregulated only in lung cancer, indicated that the transcriptomic analysis of microarray datasets allows us to find a characteristic metafirm of the tumorigenic process at the molecular level. One-hundred-forty common DEGs are downregulated and one-hundred-fifty-six overregulated in at least eight of the ten lung cancer datasets analyzed and at least one set of other types of cancer (Supplementary Table S1). Fifty-nine common DEGs are downregulated in the three types of cancer (LC&BC&LK), seventy-nine in lung cancer and breast cancer (LC&BC), and eleven in lung cancer and leukemia (LC&LK). Seventy-eight common DEGs are overregulated in the three types of cancer (LC&BC&LK), seventy-four in lung cancer and breast cancer (LC&BC), and eleven in lung cancer and leukemia (LC&LK). The ninety-eight LC winning DEGs are deregulated in at least seven of the ten sets of lung cancer, of which fifty-seven are downregulated and forty-one overregulated (Supplementary Table S2).

**Figure 2:**
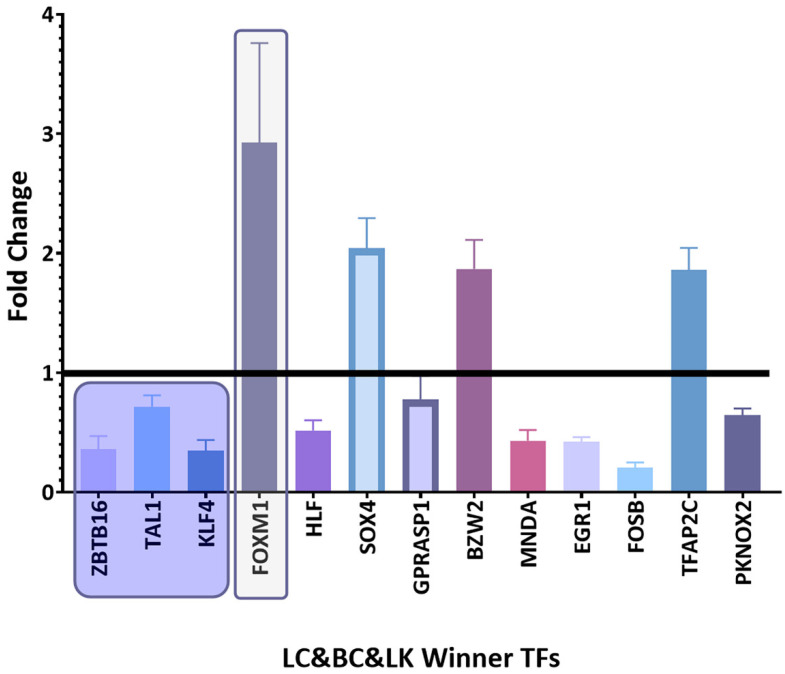
Bar graph comparing the fold change expression levels of common winning transcription factors in the three cancer types (LC&BC&LK). Transcription factors with fold change values greater than 1 are upregulated, while those with fold change values less than 1 are downregulated. Inside the boxes are the deregulated transcription factors in most datasets and those that are coexpressed in gene networks. The image was made using GraphPad Prism version 8.00 for Windows, GraphPad Software, La Jolla, California, USA, www.graphpad.com (accessed on 23 June 2022).

**Figure 3:**
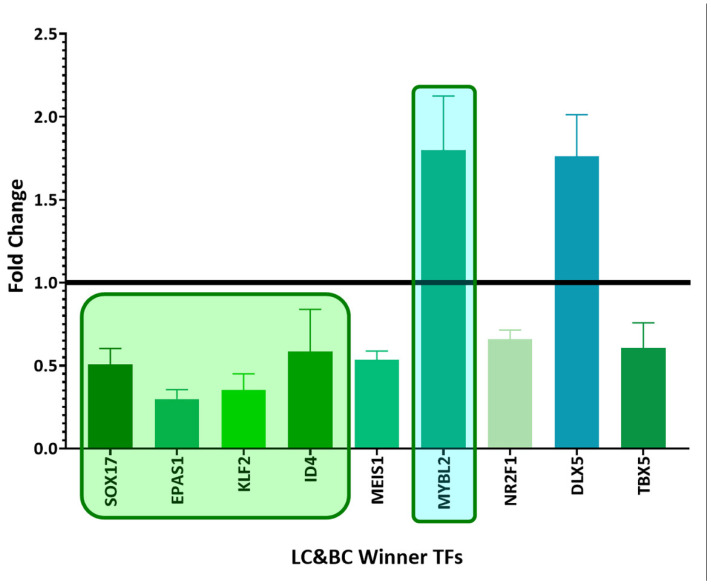
Bar graph comparing the fold change expression levels of common winning transcription factors in lung cancer and breast cancer (LC&BC). Transcription factors with fold change values greater than 1 are upregulated, while those with fold change values less than 1 are downregulated. Inside the boxes are the deregulated transcription factors in most datasets and those that are coexpressed in gene networks. The image was made using GraphPad Prism version 8.00 for Windows, GraphPad Software, La Jolla, California, USA, www.graphpad.com (accessed on 23 June 2022).

**Figure 4:**
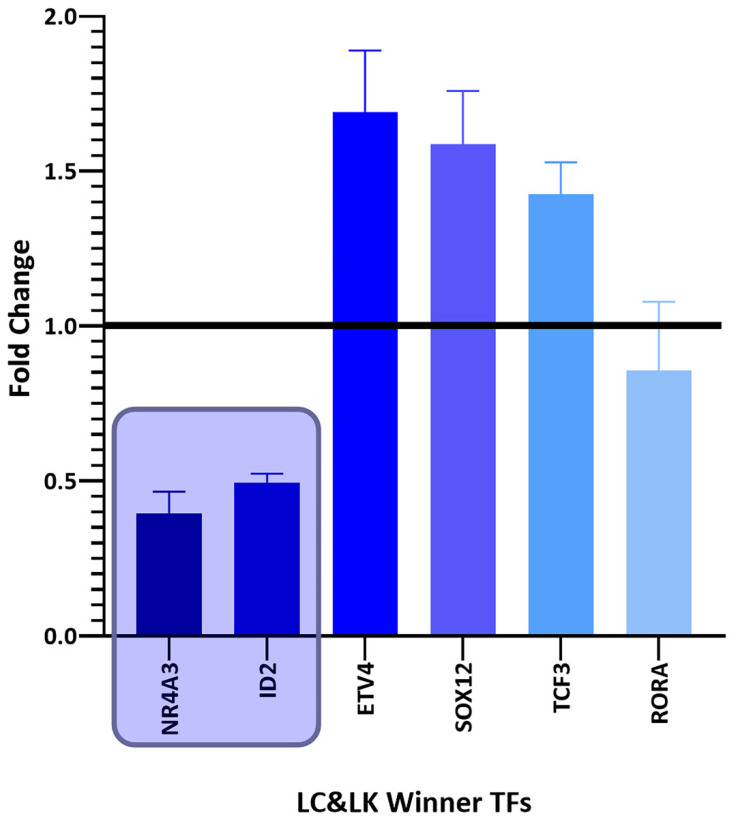
Bar graph comparing the fold change expression levels of common winning transcription factors in lung cancer and leukemia (LC&LK). Transcription factors with fold change values greater than 1 are upregulated, while those with fold change values less than 1 are downregulated. Inside the boxes are the deregulated transcription factors in most datasets and those that are coexpressed in gene networks. The image was made using GraphPad Prism version 8.00 for Windows, GraphPad Software, La Jolla, California, USA, www.graphpad.com (accessed on 23 June 2022).

**Figure 5:**
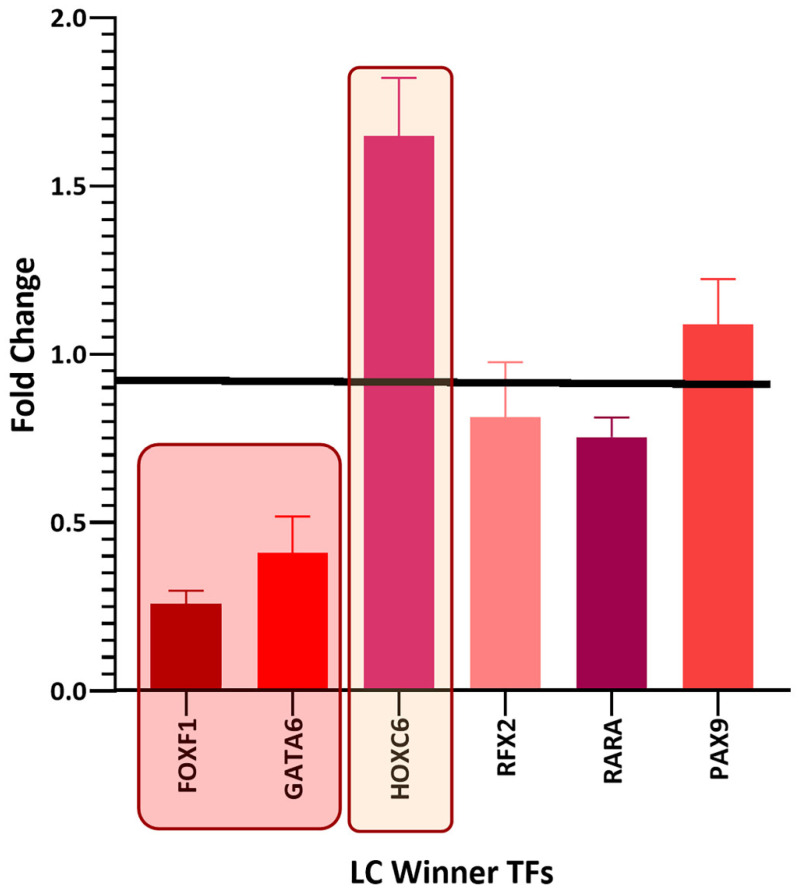
Bar graph comparing the fold change expression levels of unique lung cancer winning transcription factors in lung cancer (LC). Transcription factors with fold change values greater than 1 are upregulated, while those with fold change values less than 1 are downregulated. Inside the boxes are the deregulated transcription factors in most datasets and those that are coexpressed in gene networks. The image was made using GraphPad Prism version 8.00 for Windows, GraphPad Software, La Jolla, California, USA, www.graphpad.com (accessed on 23 June 2022).

Among the winning DEGs, there are twenty-five winning TFs common between the different types of cancer and six winning TFs unique to lung cancer (Table 2). There are thirteen deregulated winning TFs in common among the three types of cancer studied (Figure 2), nine deregulated winning TFs in common between lung cancer and breast cancer (Figure 3), and six deregulated winning TFs in common between lung cancer and leukemia (Figure 4). Additionally, there are six deregulated unique LC winning TFs (Figure 5).

### 3.2. Functional Annotation and Enrichment Analysis

**Figure 6:**
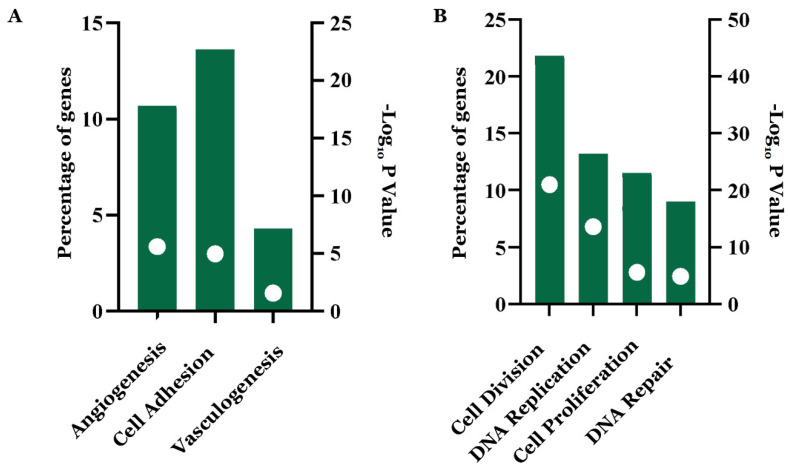
Biological processes associated with deregulated genes in common between lung cancer and other types of cancer. (**A**) Downregulated; (**B**) overregulated. The image was made using GraphPad Prism version 8.00 for Windows, GraphPad Software, La Jolla, California, USA, www.graphpad.com (accessed on 23 June 2019).

The functional annotation analysis of the 140 negatively regulated winning DEGs showed that they are associated with angiogenesis, vasculogenesis, and cell adhesion processes (Figure 6A); meanwhile, the 156 upregulated winning DEGs are associated with cell division, cell proliferation, DNA replication, and repair (Figure 6B). Likewise, 71 overregulated winning associated DEGs (*p*-value: 1.70 × 10^−12^) were identified (Supplementary Table S1). The enrichment and functional annotation analysis of the 98 unique negatively and positively regulated winning DEGs of lung cancer did not yield significant associations with cancer, biological processes, or signaling pathways (Supplementary Table S2).

According to DAVID’s analysis, sixteen winning TFs (DLX5, EGR1, FOXF1, FOXM1, GATA6, ID2, KLF2, KLF4, RARA, RORA, SOX17, SOX4, TAL1, TBX5, TCF3, and ZBTB16) are related to the positive regulation of transcription (*p*-value: 3.1 × 10^−11^), and eight winning TFs (FOXM1, GATA6, ID2, ID4, KLF4, PAX9, RARA, and ZBTB16) are related to the negative regulation of transcription (*p*-value: 4.6 × 10^−3^). Five winning TFs (EPAS1, MEIS1, RORA, SOX17, and TAL1) are related to angiogenesis (*p*-value: 3.9 × 10^−2^). Five winning TFs (KLF4, RARA, SOX4, TBX5, and ZBTB16) are associated with the negative regulation of cell proliferation (*p*-value: 1.7 × 10^−1^). Four winning TFs (FOXM1, MEIS1, RARA, and SOX4) are related to the positive regulation of cell proliferation (*p*-value: 5.8 × 10^−1^). Three winning TFs (FOXF1, KLF4, and RORA) are related to negative regulation of inflammatory response (*p*-value: 3.0 × 10^−1^). Seven winning TFs (ETV4, ID2, MEIS1, NR4A3, RARA, TCF3, and ZBTB16) are related to transcriptional misregulation in cancer (*p*-value: 6.9 × 10^−5^). Additionally, six winning TFs (DLX5, ID2, ID4, KLF4, MEIS1, and TCF3) are related to signaling pathways regulating pluripotency of stem cells (*p*-value: 1.6 × 10^−4^).

### 3.3. Coexpression Network Analysis

**Figure 7:**
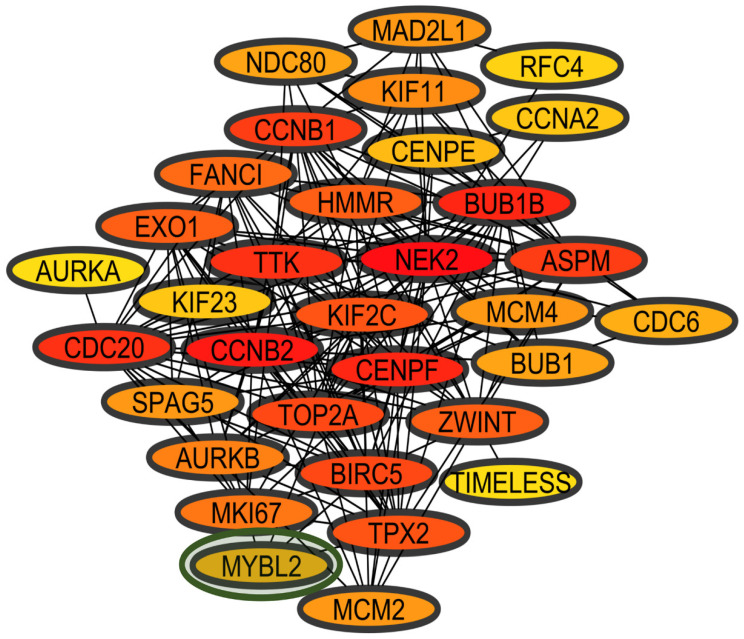
Coexpression network of deregulated winning genes common among the three types of cancer (LC&OC coexpression network). In red are the most connected nodes, in orange the average connected nodes, and in yellow the less connected nodes. The TF is highlighted inside the green oval.

The LC&OC coexpression network was created with the 71 DEGs in common between lung cancer and other types of cancer, which are associated with cancer according to the DAVID enrichment and functional annotation analysis, along with the 45 TFs identified by oPOSSUM as possible regulators of these DEGs, showing that 32 common winning DEGs are coexpressed with one TF (MYBL2) in lung cancer (Figure 7). MYBL2 is positively regulated in eight datasets of lung cancer and in two datasets of breast cancer (Table 2). Fourteen DEGs of the LC&OC coexpression network are also in the LC&LD coexpression network (the DEGs network that might be related to cancer establishment as those DEGs are also deregulated in other lung diseases), and ten DEGs are in the LCI coexpression network (the DEGs network that might be related to cancer progression as those DEGs are not deregulated in other lung diseases) [51]. Moreover, seven DEGs (AURKA, BUB1, CDC6, MAD2L1, NDC80, ZWINT, and TIMELESS) are unique to the LC&OC coexpression network (Figure 7). According to DAVID´s analysis, these seven DEGS are related to cell division (*p*-value: 5. 1 × 10^−9^).

**Figure 8:**
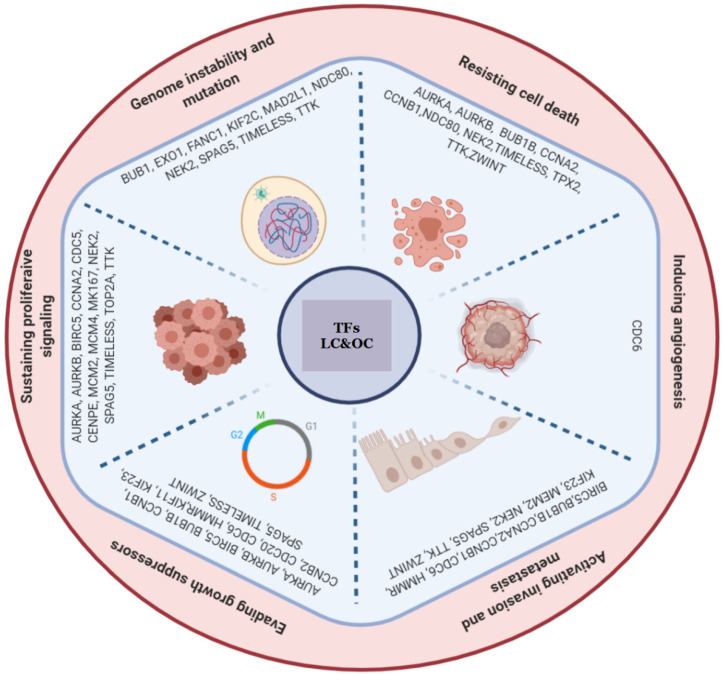
Hallmarks of cancer related to the genes of the LC&OC coexpression network.

The functional annotation analysis of the genes in the LC&OC coexpression network showed statistically significant associations to cancer (*p*-value: 5.2 × 10^−19^) and to the same biological processes and the same signaling pathways of positively regulated genes in common between lung cancer and other lung diseases, with significant *p* values (Supplementary Table S1). Most of the genes in the LC&OC coexpression network are associated with seven of the ten hallmarks of cancer (Figure 8). Most of the genes in the LC&OC coexpression network have evidence of their deregulation in lung cancer (Supplementary Table S3). Three DEGs (ASPM, CENPF, and RFC4) have no evidence of their association with the acquisition of lung cancer characteristics, and only one of them has evidence of deregulation in lung cancer (Supplementary Table S3). MYBL2 has been associated with genomic instability processes and the maintenance of proliferative signaling [52].

**Figure 9:**
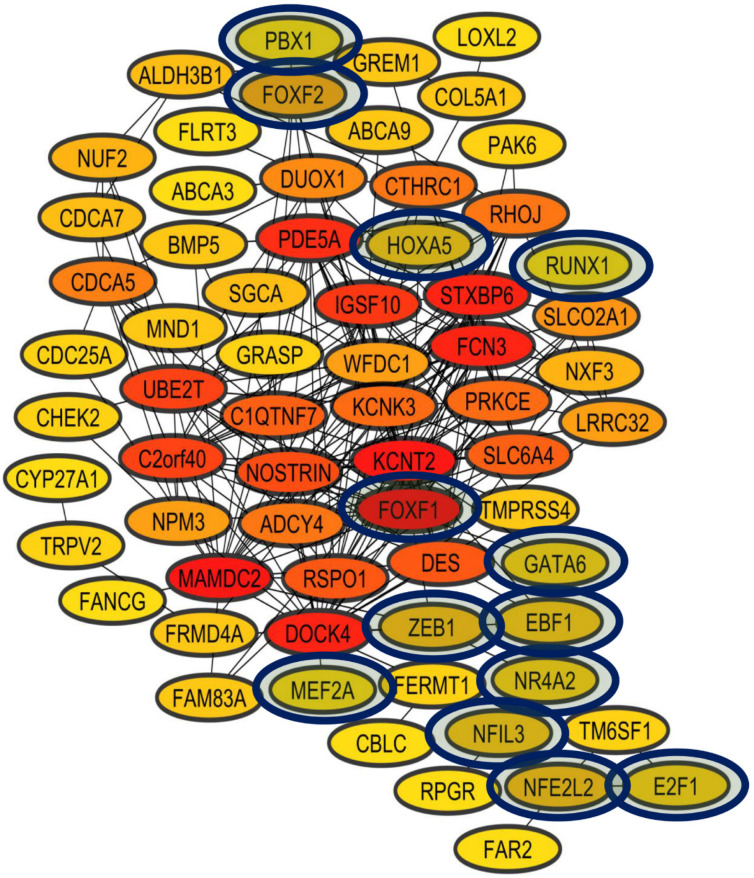
Coexpression network of unique lung cancer winning deregulated genes that are not deregulated in other types of cancer (LCII coexpression network). In red are the most connected nodes, in orange the average connected nodes, and in yellow the less connected nodes. The TFs are highlighted inside the blue ovals.

The list of the DEGs unique to lung cancer was used to make the LCII coexpression network as we could not have a list of genes related to cancer according to DAVID analysis (Supplementary Table S2), along with the 90 TFs identified by oPOSSUM as possible regulators of these DEGs. The analysis identified 53 DEGs that are coexpressed with 13 TFs in lung cancer (Figure 9). Three TFs (E2F1, NR4A2, and ZEB1) also appeared in the LC&LD coexpression network and in the LCI coexpression network, and two TFs (RUNX1 and EBF1) in the LCI coexpression network [20,51]. The other eight TFs are new and unique to the LCII coexpression network. FOXF1 is negatively regulated in eight sets of lung cancer data and in the PAH set. GATA6 is negatively regulated in seven sets of lung cancer data and in the PAH set. FOXF2 is negatively regulated in six sets of lung cancer data and in the PAH set, and positively regulated in a leukemia dataset. HOXA5 is negatively regulated in six sets of lung cancer data, in two of breast cancer, and in the PAH set. MEF2A is negatively regulated in four sets of lung cancer and in the PAH set, and positively regulated in one set of breast cancer and in two of leukemia. NFE2L2 is positively regulated in two sets of lung cancer data, one set of leukemia, and in the PAH set, and negatively regulated in three sets of lung cancer, two of breast cancer, and in one of leukemia [20]. NFIL3 is negatively regulated in four sets of lung cancer and two sets of leukemia. PBX1 is negatively regulated in two sets of lung cancer and two sets of leukemia, and positively regulated in a set of breast cancer.

**Figure 10:**
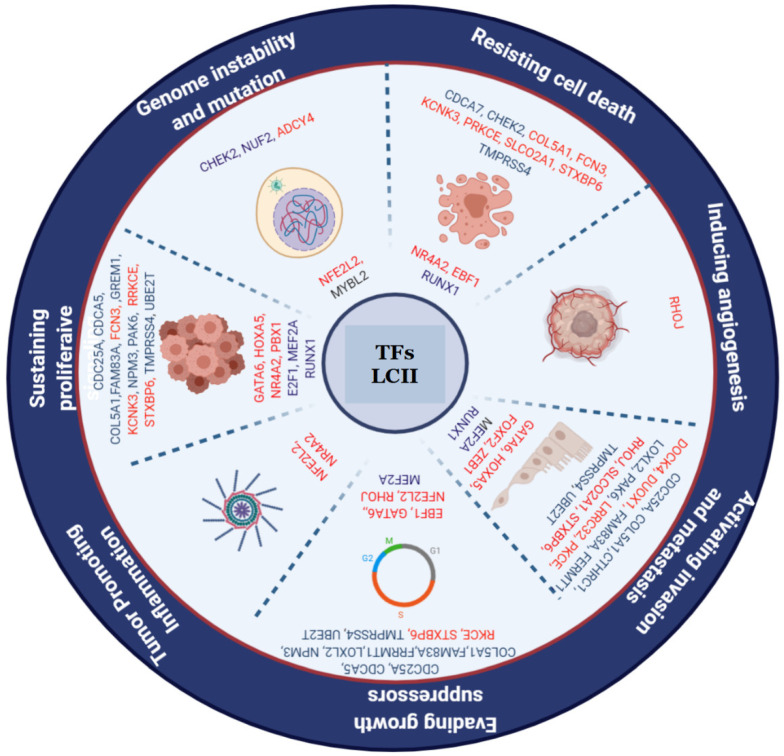
Hallmarks of cancer related to the genes of the LCII coexpression network. The TF is in the center of the circle, and the DEGs are more external in the circle. All overregulated genes are in blue, and all downregulated genes are in red.

Half of the genes in the LCII coexpression network have experimental evidence of their association with seven of the ten hallmarks of cancer (Figure 10). In the LCII coexpression network, 18 DEGs (ABCA3, ALDH3B1, C1QTNF7, CBLC, CYP27A1, DES, FANCG, FR, FLRT3, MD4A, IGSF10, KCNT2, MAMDC2, MND1, PDE5A, RSPO1, SLC6A4, TM6SF1, and W6DC) have evidence of their deregulation in lung cancer but have not yet been associated with the acquisition of any hallmark of cancer (Supplementary Table S4). The expression of four DEGs (C2orf40, NOSTRIN, NFX3, and TRPV2) has been associated with normal lung function, but they have no experimental evidence of their deregulation in cancer, or of their association with the acquisition of the hallmarks of cancer. Five DEGs (ABCA9, FAR2, GRASP, RPGR, and SGCA) have no evidence of their deregulation in lung cancer, nor any experimental evidence of their association with the acquisition of the hallmarks of cancer. NFIL3, a TF, also has no evidence of its deregulation in lung cancer, nor any experimental evidence of its association with the acquisition of the hallmarks of cancer (Supplementary Table S4).

The comparison of lung cancer (LC) and breast cancer (BC) coexpression networks with Gedevo identified five statistically significant alignments (Median > 0.5), appearing in at least 6.3% of all possible alignments (Table 3). The genes associated with the LC-BC alignments are new genes; none had appeared in any of the previous coexpression analyses. Four of the five genes (HEG1, PLSCR4, GMFG, and NME4) associated with LC-BC alignments have been observed to be deregulated in lung cancer. ReactomeFIViz enrichment analysis in Cytoscape showed that two genes (HEG1 and PLSCR4) have evidence of their association with the acquisition of cancer characteristics.

The comparison of lung cancer (LC) and leukemia (LK) coexpression networks with Gedevo identified seven statistically significant alignments (Median>0.5), appearing in at least 7.5% of all possible alignments (Table 4). Six of the genes associated with the LC-LK alignments are new genes; only one (BIRC5) appears in the LC&LD coexpression network. Five of the seven genes (BIRC5, GIMAP5, HBB, IL33, and AKAP12) associated with the LC-LK alignments have been observed to be deregulated in lung cancer. ReactomeFIViz enrichment analysis in Cytoscape showed that four genes (SNRK, BIRC5, HBB, and IL33) have evidence of their association with the acquisition of cancer characteristics.

The Coexnet library found some CCPs in some comparisons of lung cancer (LC) and other types of cancer (OC), the majority made of two or three nodes (Supplementary Table S5). The GSE10072 lung cancer set formed the largest CCPs with two breast cancer (BC) sets; the GSE3744 set formed a six-node CCP, and the GSE21422 set formed a 12-node CCP and a six-node CCP. A total of 44 nodes were found in the different CCPs identified by Coexnet when comparing the coexpression networks of LC and BC. The analysis of the nodes of the LC-BC CCPs with iRegulon showed SOX15 as a regulator of 26 of the 44 nodes with an enrichment score of 4712 and 13 possible binding motifs. SOX15 is positively regulated in four sets of lung cancer and in one of leukemia, and negatively regulated in a set of breast cancer. ReactomeFIViz enrichment analysis in Cytoscape showed an association of seven (EDNRB, CDKN2A, VEGFD, FOS, GNG11, TGFBR2, and BIRC5) of the forty-four nodes of the LC-BC CCPs with signaling pathways related with the acquisition of tumor characteristics.

The GSE10072 lung cancer set formed the majority of CCPs with four sets of leukemia (LK), with two or three nodes in total each. A total of twelve nodes appeared in the different CCPs identified by Coexnet when comparing the coexpression networks of lung cancer (LC) and leukemia (LK). The analysis of the nodes with iRegulon showed HLF, a TF with the ability to regulate four nodes, has an enrichment score of 6000, has six possible binding motifs, and is negatively regulated in eight sets of lung cancer data. ReactomeFIViz enrichment analysis in Cytoscape showed an association of two nodes (CAV1 and TNF) with the TNF receptor signaling pathway, and another two nodes (FOSB and TNF) with the IL−7 signaling pathway.

### 3.4. Coregulatory Network Analysis

According to Coregnet´s analysis, in lung cancer, 21 winning TFs (ZBTB16, ID2, ID4, EPAS1, EGR1, FOSB, HLF, FOXF1, GATA6, GPRASP1, KLF2, MEIS1, MNDA, NR4A3, TAL1, RFX2, RORA, SOX17, PKNOX2, NR2F1, and KLF4) can form a coregulatory network, and nine winning TFs (MYBL2, FOXM1, HOXC6, BZW2, TCF3, SOX4, ETV4, SOX12, and TFAP2C) can form another coregulatory network. In breast cancer, eight winning TFs (ZBTB16, KLF2, KLF4, NR2F1, EGR1, FOSB, EPAS1, and GPRASP1) can form a coregulatory network, and three other winning TFs (MYBL2, FOXM1, and TAL1) can form another coregulatory network. In leukemia, there is no evidence that the winning TFs have the ability to form any coregulatory complex.

### 3.5. Survival Analysis of Top Winning Transcription Factors in Lung Cancer

**Figure 11:**
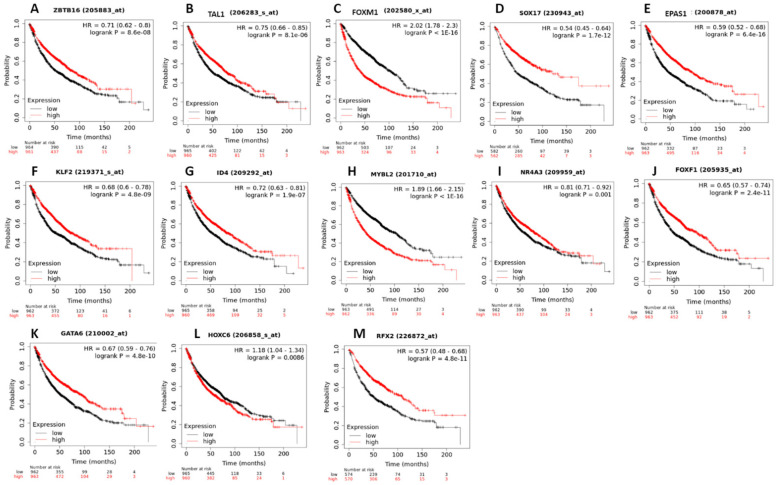
KM plots of the top winning transcription factors associated with lung cancer patients’ survival, and with a significant logrank *p*-value. (**A**) ZBTB16, (**B**) TAL1, (**C**) FOXM1, (**D**) SOX17, (**E**) EPAS1, (**F**) KLF2, (**G**) ID4, (**H**) MYBL2, (**I**) NR4A3, (**J**) FOXF1, (**K**) GATA6, (**L**) HOXC6, (**M**) RFX2.

The KM plotter analysis for the top winning TFs showed a statistically significant association between the expression levels of ZBTB16, TAL1, FOXM1, SOX17, EPAS1, KLF2, ID4, MYBL2, NR4A2, FOXF1, GATA6, HOXC6, and RFX2 with the survival of lung cancer patients (Figure 11).

## 4. Discussion

**Figure 12:**
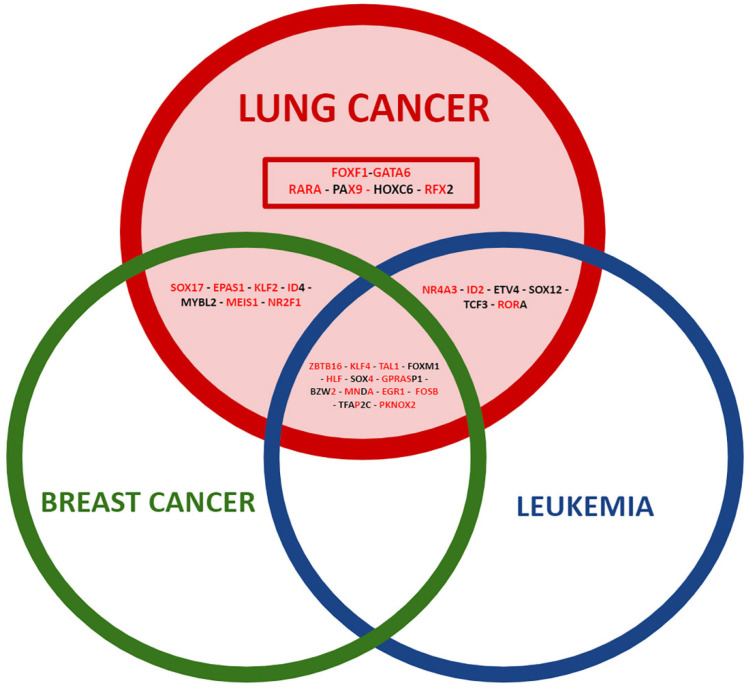
Venn diagram with the transcriptomic metafirm of winning and coexpressed transcription factors common among the three types of cancer and unique to lung cancer. Negatively regulated transcription factors are in bold red, and positively regulated transcription factors are in bold black.

The search for transcription factors as general and specific tumor biomarkers of lung cancer began with the selection of an important number of comparable datasets, from which we could identify and establish the most complete transcriptome metafirm possible of the tumor process independent of tissue and specifically in the lung (Figure 12), taking into account different populations and all possible types and subtypes of cancer, in order to select the most accurate list of common deregulated genes in leukemia, lung, and breast cancer, and an important number of lung cancer studies to identify those unique deregulated genes. We have chosen epidemiologically important cancer types in two of our population groups (breast cancer in women and leukemia in children), as well as one type of cancer with an embryonic origin close to the lung (breast cancer) and a type with a different embryonic origin (leukemia). Therefore, we could expect to have a greater number of genes in common with the type of cancer with a closer embryonic origin if there is some relationship between the transcriptional regulatory processes during embryonic and tumor stages in different tissue types, as well as to have a bigger picture of the general tumoral processes with different embryonic origins. Indeed, we found a greater number of DEGs (Supplementary Table S1) and winning TFs (Table 2) between lung cancer and breast cancer compared to lung cancer and leukemia. Therefore, it was also possible to identify unique and common genes regardless of the great heterogeneity of tumor diseases in terms of cell or tissue type, and different types and subtypes of cancer.

When comparing the three types of cancer, we found thirteen TFs within at least ten datasets (Table 2). The top four winning TFs are ZBTB16, TAL1, KLF4, and FOXM1. ZBTB16 (zinc finger and BTB domain containing 16) is a TF involved in key developmental processes, self-renewal, and differentiation of stem cells. ZBTB16 is downregulated in lung cancer, breast cancer, and leukemia, is the first winning TF of the three types of cancer, and, therefore, is a potential general tumor biomarker. ZBTB16 is a member of the Krüppel C2H2-type zinc-finger protein family of TFs, which has been found to be upregulated in clear cell renal cell carcinoma, colon cancer, glioblastoma, testicular seminoma, and downregulated in hepatocellular carcinoma, lung cancer, melanoma, pancreatic cancer, prostate cancer, and thyroid carcinoma [53]. The downregulation of ZBTB16 in the cytoplasm of NSCLC lung cancer cells has been related to high tumor grade and tumor aggression [54]. In breast cancer, ZBTB16 is also downregulated by promoter hypermethylation, and, when activated, it can inhibit breast cancer cells’ proliferation and metastasis [55]. Therefore, ZBTB16 has shown tumor suppression activity in cancer.

TAL1 (T-cell acute lymphocytic leukemia protein 1) is a basic-helix-loop-helix (bHLH) TF [56], an essential regulator of normal hematopoiesis [57], and also a winning downregulated TF in three types of cancer. TAL1 is a hub node of a transcriptional regulatory network in lung adenocarcinoma, promoting the TGF-β signaling pathway [58]. KLF4 (Krüppel-like factor 4) is a zinc finger-type TF important during development, differentiation, and tissue homeostasis [59]. KLF4 is downregulated in colorectal carcinoma [60], and gastric epithelium [61], suggesting that it might be a tumor suppressor. FOXM1 (Forkhead box transcription factor) is related to cell cycle, cell differentiation and proliferation, DNA damage repair, tissue homeostasis, angiogenesis, apoptosis, and metastasis [62]. FOXM1 is highly expressed in several cancers, such as hepatocellular carcinomas [63], colon cancer [64], pancreatic cancer [65], gastric cancer [66], and breast cancer [67]. We have also identified FOXM1 as an important regulator during lung cancer progression since it is the TF that regulates a greater number of genes deregulated only in lung cancer, which are not deregulated in other lung diseases and are coexpressed in the common connectivity patterns formed between the gene networks of lung cancer datasets [20].

When comparing lung cancer and breast cancer, we found nine TFs within at least nine datasets (Table 2). The top four winning TFs are SOX17, EPAS1, KLF2, and ID4. SOX17 (SRY-box containing gene 17) is a member of the SRY-related high mobility group (HMG) box family of TFs, related to embryogenesis and the negative modulation of the WNT/β-catenin and TCF signaling pathway in lung cancer [68]. SOX17 is downregulated by promoter hypermethylation, contributing to the activation of the Wnt signaling pathway in breast cancer establishment and progression [69]. SOX17 is downregulated in lung and breast cancer, is the first winning TF of these two types of cancer, and is, therefore, a potential general tumor suppressor biomarker related to ectoderm origin [70]. EPAS1 (endothelial PAS domain-containing protein 1) is downregulated in NSCLC due to promoter methylation and sequence genetic polymorphisms [71,72], and specifically in lung adenocarcinoma [73]. EPAS1 is negatively regulated by miR−152–3p, which induces apoptosis of MCF−7/TAX cells [74]. KLF2 (Kruppel-like factor 2) binds to GC-enriched promoter regions of genes involved in apoptosis [75] and inhibition of angiogenesis [76], proliferation, and migration [77]. In NSCLC, KLF2 is downregulated and has a tumor suppressor function [78]. KLF2 is also downregulated in breast cancer, having a tumor suppressor activity that can control the transcriptional activity of vitamin A metabolite retinoic acid (RA), while its expression positively correlates with patients’ survival [79]. ID4 (inhibitor of DNA binding 4) is a tumor suppressor that inhibits epithelial–mesenchymal transition and metastasis [80] and induces apoptosis in lung adenocarcinoma through the activation of the p38 MAPK signaling pathway [81]. However, the expression of ID4 is negative in breast cancer, NSCLC, and its subtype datasets, but it is only positive in the SCLC dataset, which suggests a difference in expression levels according to the type of lung cancer. This may suggest that the function of this TF varies depending on the type of lung cancer, and, therefore, its function could be that of a tumor suppressor in NSCLC and that of an oncogene in SCLC.

When comparing lung cancer and leukemia, we found nine TFs within at least nine datasets (Table 2). The top two winning TFs are NR4A3 and ID2. NR4A3 (nuclear receptor subfamily 4, group A, member 3) is an early immediate gene considered a possible homeostatic regulator of proliferation, apoptosis and differentiation, and tumor suppressors in rapidly lethal acute myeloid leukemia (AML) [82]. NR4A3 acts as a tumor suppressor in lung and breast cancer, favoring the activation of programmed cell death programs [83]. ID2 (DNA binding protein inhibitor 2) is a helix-loop-helix TF that positively regulates cancer cells’ proliferation [84], migration, invasion [85], and cell cycle progression, and also negatively regulates cancer cells’ differentiation and apoptosis [86], as well as other tumor suppressor genes [87]. ID2 has a role in the dedifferentiation of NSCLC cells, suggesting that it can be used as a prognostic marker [88].

On the basis assumption that coexpression implies coregulation, the identification of TFs as hub genes in coexpression modules that are likely to be the regulators of gene coexpression can be made through a transcriptomic analysis in several cancer types and subtypes, which may share genetic components and expression patterns, to identify a gene coexpression and coregulatory metafirm of TFs that represent an architecture of general tumors and those specific to lung cancer [89]. The construction of a coexpression network with common deregulated winning DEGs between three types of cancer (LC&OC network) allowed the identification of the coexpressed DEGs and TFs that can be more important for the establishment and progression of any tumor pathology (Figure 7). The LC&OC network has 15 DEGs also coexpressed in the LC&LD network associated with the same hallmarks of cancer, suggesting that slightly more than half of the DEGs of the LC&LD network are associated with the establishment of tumor pathology specifically in the lung.

The seven DEGs (AURKA, BUB1, CDC6, MAD2L1, NDC80, ZWINT, and TIMELESS) coexpressed only in the LC&OC coexpression network have been associated with the acquisition of the hallmarks of cancer. AURKA is overexpressed in poorly differentiated lung cancer cells [90]. AURKA is positively regulated by KRAS, but, if it is negatively regulated, it is related with decreases in growth, viability, transformation, proliferation, and apoptosis [91]. CDC6 is negatively regulated by miR26a and miR26b, decreasing proliferation and metastasis of lung cancer cells [92]. CDC6 is regulated by E2F TFs [93,94]. CDC6 reduces levels of E-cadherin [95]. Ang-(1-7) inhibits CDC6, decreasing cell growth and EMT [96]. The Leu84Met SNP of MAD2L1 has an increased related risk to progress into lung cancer depending on the allele dose [97]. NEK2 is degraded after inhibition of its interaction with Hec1, leading to induction of chromosomal misalignment in metaphase and apoptosis [98]. The negative regulation of TIMELESS inhibits proliferation, growth, and induces apoptosis [99]. The negative regulation of ZWINT reduces proliferation, migration, invasion, and apoptosis [100].

In the LC&OC coexpression network, there are three DEGs (ASPM, CENPF, and RFC4) that have no evidence of their association with the acquisition of lung cancer characteristics, and only one of them has evidence of its deregulation in lung cancer (Supplementary Table S3). CENPF also appears in the LCI coexpression network and is one of the genes that has been deregulated in lung cancer but has not been associated with the acquisition of the hallmarks of cancer in this tissue. In the LC&OC network, eleven DEGs of the LCI network were also coexpressed, suggesting that most of the LC&OC network DEGs and MYBL2 are associated with the progression of tumor pathology. Since it has been suggested that other diseases may be the origin of tumor processes in the lung [101], therefore the deregulated genes in both lung cancer and lung diseases may be associated with the establishment of tumor pathology, while the genes deregulated only in lung cancer may be associated with the progress of cancer [20,51]. In general, MYBL2 has been expressed in proliferating cells [102], and its ability to maintain adequate proliferative signaling is considered necessary to maintain genomic stability [103]. However, MYBL2 has shown the ability to induce proliferation and cell cycle in lung adenocarcinoma [104]. Although the different types of cancer show the deregulation of a large number of common winning DEGs, there is only one TF coexpressed in the LC&OC network. Likewise, the LC&OC CCPs are very small compared to the previously identified LC&LD CCPs, and they show new associations not observed in the coexpression networks of cancer-associated winning DEGs, which might demonstrate the great specificity and complexity of cancer since it suggests that different TFs are involved with a large number of very different deregulated genes in the establishment and progression of tumor pathology in each tissue.

The LC-BC CCPs formed have no more than 12 nodes and identified only seven genes (EDNRB, CDKN2A, VEGFD, FOS, GNG11, TGFBR2, and BIRC5) compromised in signaling pathways associated with the acquisition of tumor characteristics. The LC-LK CCPs are made of two or three nodes maximum and identified four genes (SNRK, BIRC5, HBB, and IL33) associated with the acquisition of the hallmarks of cancer (Supplementary Table S5). BIRC5 is a gene identified in the analysis as a DEG associated with the establishment of cancer in the LC&LD and LC&OC networks, and its presence in the LC-BC and LC-LK CCPs suggests its importance in tumor process. The overexpression of BIRC5 can lead to cell cycle activation to promote the development of lung adenocarcinoma, while negative regulation can dramatically decrease invasion and metastasis capabilities, suppress proliferation, slow growth, induce vascular pulmonary apoptosis, and reverse pulmonary arterial hypertension [105]. The other seven genes (EDNRB, CDKN2A, VEGFD, GNG11, SNRK, HBB, and IL33) do not appear in the analysis of coexpression networks of winning DEGs; they are new genes with evidence of their association to the acquisition of the characteristics of cancer that must be studied to know its importance in tumor processes of different tissues (Supplementary Table S5).

The analysis of the LCII coexpression network allowed us to identify a significant number of unique lung cancer genes that are not deregulated in other types of cancer. Unique lung-cancer-winning DEGs that are coexpressed in the LCII network are new; they do not appear in the three coexpression networks of previous cancer-associated winning DEGs, suggesting their importance in the establishment and progression of cancer specifically in lung tissue (Figure 9). More than half of the DEGs of the LCII network should be studied experimentally because they do not have specific evidence of their deregulation in lung cancer, or their association with the acquisition of the hallmarks of cancer. The DEGs that have experimental evidence have been associated with invasion, metastasis, sustained proliferative signaling, evasion of growth suppressors, resistance to cell death, and genomic instability, the same hallmarks associated with the three previous networks with the winning DEGs associated with cancer (Supplementary Table S4). The study of the other half of DEGs can strengthen the hallmarks already identified in the analysis, increasing the number of winning DEGs associated with each of them, and it can highlight the importance of other hallmarks of cancer during the establishment and progression of lung cancer.

The LCII coexpression network allowed to identify a group of TFs that further strengthened the TF regulation network identified with the transcriptomic and coexpression analysis of lung cancer and other lung diseases [20]. E2F1, NR4A2, and ZEB1 reappear coexpressed in the LCII network, further verifying their importance for the establishment and progression of lung cancer since they are also in the LC&LD network and in the LCI network. Likewise, EBF1 and RUNX1 also appear in the LCII network, and they are also in the LCI coexpression network, probably strengthening their importance and association with the progression of some types of lung cancer [51]. EBF1 is only deregulated in lung cancer, but only in two of the ten datasets, while RUNX1 is deregulated in lung cancer and breast cancer, but its regulation varies: it is positive in some datasets and negative in others of the same type of cancer. The RUNX family of TFs can act as an oncogene or as a tumor suppressor during oncogenic processes, suggesting their importance as biomarkers of cancer [106]. The other eight TFs are unique to the LCII coexpression network, which suggests that they are more important for lung cancer than for other types of cancer as they do not appear in the previous networks. Moreover, their association with the establishment or progression should be studied experimentally, along with the enrichment of their binding motifs in the winning DEGs associated with lung cancer.

The transcriptomic analysis (Supplementary Table S2) and the experimental evidence available for each of the TFs of the LCII network (Supplementary Table S4) showed that six TFs of the LCII network are deregulated in between three and six sets of lung cancer, and also in some other types of cancer. FOXF1 and GATA6 stand out since they are deregulated in the majority, eight and seven, respectively, of the ten sets of lung cancer, in none of the others types of cancer (Table 2), and in the PAH set, which suggests their association with the establishment of tumor pathology in the lung [20]. Experimental studies have shown that the negative regulation of FOXF1 is related to the expression of genes associated with extracellular matrix remodeling and cell cycle progression during the regeneration of lung tissue [107]. Likewise, FOXF1 has proven to be a mediator of cellular reprogramming to reacquire stem characteristics [108]. Long non-coding RNA located near the coding region of FOXF1, called FOXF1-AS1, has been negatively regulated in lung cancer [109], associated with EMT, cell reprogramming, metastasis [110], and growth inhibition in NSCLC cancer cells [111]. On the other hand, retinoic acid affects the growth of lung adenocarcinoma by inducing cell differentiation and inhibiting proliferation after the activation of GATA6, and the inhibition of Wnt and EGFR [112]. GATA6 is essential for lineage selection, which directly associates effectors for lung epithelium specification and the inhibition of metastasis in lung adenocarcinoma [113].

HOXC6 and RFX2 are also deregulated in eight sets of lung cancer as FOXF1 and none of the other types of cancer (Table 2). HOXC6 (homeobox protein Hox-C6) is an overexpressed TF with a very high oncogenic potential in NSCLC progression as it is a regulator of genes related to cancer cells proliferation and metastasis [114]. RFX2 (regulatory factor X2) is overregulated in small-cell lung cancer, and it is related to chemoresistance [115]. The expression of RFX2 is negative in NSCLC and its subtypes’ datasets but positive in the SCLC dataset, which suggests its function could be that of a tumor suppressor in NSCLC and that of an oncogene in SCLC. SOX4 (SRY-related HMG-box 4) is related to cell fate differentiation and determination in male testis fertility [116], as well as cancer development and progression [117]. SOX4 is overregulated in all ten lung cancer datasets (Table 2), suggesting that it could be a key oncogene function in lung cancer. However, SOX4 is also upregulated in ductal carcinoma in situ and invasive breast carcinoma but downregulated in two sets of chronic lymphocytic leukemia (Table 1 and Table 2). Therefore, its role could be as a tumor suppressor in leukemia and as an oncogene in lung and breast cancer.

The coregulatory analysis allowed us to identify two coregulatory networks in lung and breast cancer. The small coregulatory network has two winning TFs (FOXM1 and MYBL2) coexpressed in the gene networks that coexpressed with the common winning DEGs between the three types of cancer and the winning DEGs related to lung cancer. The big coregulatory network has the top winning TFs. There is already some evidence that the coexpressed and winning TFs can form coregulatory complexes to specify patterns of gene expression. MYBL2 forms a complex with MuvB to increase the specificity of binding of FOXM1 to its target genes. Moreover, the MMB-FOXM1 complex regulates cell cycle genes during the G2/M mitosis phase [118]. ZBTB16 makes large regulatory complexes with other molecules to bind to the regulatory elements in the promoter region of the target genes [119]. ZBTB16 binds to corepressors and histone modification enzymes to change chromatin architecture and accessibility [120]. ZBTB16/PLZF forms a rare fusion with RARA by a reciprocal chromosomal translocation t(11;17)(q23;q21) in acute promyelocytic leukemia [121]. TAL1 also forms coregulatory complexes with LDB1, LMO2 [56], and binds to coactivators or corepressor proteins to positively and negatively regulate transcription [122]. FOXF1 interacts with PRC2 to regulate gene expression [123]. FOXF1 is a part of the Fanconi anemia protein complexes to respond to damages in the DNA [124]. The Kaplan–Meier survival analysis of top winning TFs showed an important association of their deregulation with overall survival of lung cancer patients with significant log-rank p values (Figure 11), therefore suggesting that this TF network is very important for cancer establishment and progression. However, it is important to carry out in-depth studies on the regulatory complexes that these TFs can form in order to clearly identify their architecture and specificity in the regulation of gene expression in signaling pathways related to the acquisition of the hallmarks of cancer.

## 5. Conclusions

The search for TFs biomarkers associated with the establishment and progression of lung cancer was extended with the comparisons of microarray datasets of lung cancer with other types of cancer. In the present study, we applied our own bioinformatic pipeline to analyze the complex regulatory mechanisms associated with the tumoral process, establishing simple rules that could be applied in every case. First, we identified all the deregulated genes associated in the three types of cancer and specifically with lung cancer establishment or progression and, between them, we highlighted the most important transcriptional regulators, as they were coexpressed in networks, with the common and unique DEGs related to lung cancer establishment and progression and potentially being able to form coregulatory complexes.

The LC&OC network allows identifying coexpressed winning DEGs and a TF associated with the establishment and progression of the tumor process in cancer. Meanwhile, the LCII network allowed us to identify new genes and TFs associated with the acquisition of cancer characteristics specifically in lung tissue. However, the winning DEGs identified in the LCII coexpression network must be further investigated to properly associate them with the acquisition of the hallmarks of cancer during the establishment and progression of tumoral processes in lung cells. The TF network identified has experimental evidence of its association with important biological processes and signaling pathways during the acquisition of the hallmarks of cancer, thus generating the characteristic metafirm in gene expression in general tumors and those specific to lung cancer and taking into account the great heterogeneity of cancer at cellular and population levels. Therefore, the winning TF network might be related to the formation of modular constructs with cis elements, such as enhancers, in the promoter of the winner DEGs, binding simultaneously with other cofactors and/or TFs as multiprotein complexes, to finally provide cells the ability to acquire the hallmarks of cancer. The network of winning TFs represents the transcriptional metafirm of cancer since it has evidence of coexpression with winning deregulated genes in addition to the possible formation of coregulatory complexes associated with the control of tumor suppressor and oncogenic gene expression programs and, therefore, may be related to tumor establishment and progression, as well as the survival of cancer patients.

## Figures and Tables

**Table 1:** Analyzed datasets, each study code, subjects’ disease, and number of samples.

Study Code	Subjects’ Disease	Samples	Reference
GSE19804	Non-smoking women with NSCLC	Normal (60) vs. Cancer (60)	[23]
GSE10072	Patients with lung adenocarcinoma	Normal (49) vs. Cancer (58)	[24]
GSE3268	Patients with squamous lung cancer cells	Normal (5) vs. Cancer (5)	[25]
GSE108055	Typical and atypical carcinoid, and SCLC	Normal (9) vs. Cancer (54)	[26]
E-MTAB−5231	Patients with NSCLC	Normal (18) vs. Cancer (SCC = 11 AC = 11)	[27]
E-MTAB−3950	Pre-invasive and invasive early squamous carcinoma	Normal (30) vs. Cancer (30) (SCC)	[28]
GSE52248	Patients with lung adenocarcinoma	Normal (6) vs. Cancer (12)	[29]
GSE70089	Patients with lung carcinoma	Normal (3) vs. Cancer(3)	[30]
GSE81089	Patients with NSCLC	Normal (19) vs. Cancer (199)	[31]
GSE84776	Patients with squamous lung cancer cells	Normal (9) vs. Cancer (9)	[32]
GSE10797	Invasive breast cancer	Normal (10) vs. Cancer (56)	[33]
GSE21422	Ductal carcinoma in situ and invasive breast carcinoma	Normal (5) vs. Cancer (14)	[34]
GSE26910	Invasive breast primary breast cancer	Normal (6) vs. Cancer (6)	[35]
GSE3744	Sporadic basal-like breast cancer	Normal (7) vs. Cancer (40)	[36,37]
GSE5764	Invasive lobular and ductal breast cancer	Normal (10) vs. Cancer (20)	[38]
GSE22529	Chronic lymphocytic leukemia	Normal (11) vs. Cancer (41)	[39]
GSE26725	Chronic lymphocytic leukemia	Normal (5) vs. Cancer (12)	[40]
GSE5788	T-cell prolymphocytic leukemia	Normal (8) vs. Cancer (6)	[41]
GSE6691	Chronic lymphocytic leukemia	Normal (13) vs. Cancer (11)	[42]
GSE9476	Acute myeloid leukemia	Normal (38) vs. Cancer (26)	[43]

**Table 2:** List of winning transcription factors in common among the three types of cancer and unique to lung cancer. Number of deregulated lung cancer, breast cancer, and leukemia datasets.

Transcription Factors (TFs)	Lung Cancer (LC)	Breast Cancer (BC)	Leukemia (LK)	Total
ZBTB16	9	4	3	16
KLF4	9	4	1	14
TAL1	9	3	2	14
FOXM1	9	2	1	12
BZW2	9	1	1	11
HLF	8	3	1	12
GPRASP1	8	2	2	12
MNDA	8	2	1	11
PKNOX2	8	1	1	10
TFAP2C	8	1	1	10
SOX4	10	1	2	13
EGR1	7	4	3	14
FOSB	7	4	1	12
SOX17	10	3	0	13
EPAS1	8	3	0	11
KLF2	8	3	0	11
ID4	8	3	0	11
MEIS1	8	2	0	10
MYBL2	8	2	0	10
NR2F1	8	2	0	10
DLX5	8	1	0	9
TBX5	8	1	0	9
NR4A3	8	0	1	9
ID2	7	0	3	10
ETV4	7	0	1	8
SOX12	7	0	1	8
TCF3	7	0	1	8
RORA	6	0	2	8
FOXF1	8	0	0	8
HOXC6	8	0	0	8
RFX2	8	0	0	8
GATA6	7	0	0	7
RARA	7	0	0	7
PAX9	7	0	0	7

**Table 3:** Gedevo’s most significant alignments between lung cancer (LC) and breast cancer (BC) sets.

Alignment LC&BC	Number of Alignments	Percentage	Median
HEG1—HEG1	5	10.4%	0.58
PLSCR4—PLSCR4	5	10.4%	0.50
GMFG—GMFG	4	8.3%	0.51
FCGR3B—FCGR3B	3	6.3%	0.52
NME4—NME4	3	6.3%	0.50

**Table 4:** Gedevo’s most significant alignments between lung cancer (LC) sets and leukemia (LK) sets.

Alignment LC&LK	Number of Alignments	Percentage	Median
SNRK—SNRK	6	15.0%	0.51
BIRC5—BIRC5	5	12.5%	0.57
GIMAP5—GIMAP5	3	7.5%	0.51
HBB—HBB	3	7.5%	0.51
IL33—IL33	3	7.5%	0.52
AKAP12—AKAP12	3	7.5%	0.52
CD93—CD93	3	7.5%	0.60

## Data Availability

All microarray datasets are fully available in Gene Expression Omnibus (GEO) and Array Express.

## Supplementary Materials

The following supporting information can be downloaded at: https://www.mdpi.com/article/10.3390/biology11071082/s1, Table S1. Winner overregulated and downregulated DEGs in common between lung cancer and other types of cancer. Table S2. Winner overregulated and downregulated DEGs in lung cancer that are not deregulated in other types of cancer. Table S3. Experimental evidence of the LC&OC network genes related to the hallmarks of cancer. Table S4. Experimental evidence of the LCII network genes related to the hallmarks of cancer. Table S5. Overregulated and downregulated DEGs in CCP analysis of lung cancer and other types of cancer. References [125,126,127,128,129,130,131,132,133,134,135,136,137,138,139,140,141,142,143,144,145,146,147,148,149,150,151,152,153,154,155,156,157,158,159,160,161,162,163,164,165,166,167,168,169,170,171,172,173,174,175,176,177,178,179,180,181,182,183,184,185,186,187,188,189,190,191,192,193,194,195,196,197,198,199,200,201,202,203,204,205,206,207,208,209,210,211,212,213,214,215,216,217,218,219,220,221,222,223,224,225,226,227,228,229,230,231,232,233,234,235,236,237,238,239,240,241,242,243,244,245,246,247,248,249,250,251,252,253,254,255,256,257,258,259,260,261,262,263,264,265,266,267,268,269,270,271,272,273,274,275,276,277,278,279,280,281,282,283,284,285,286,287,288,289,290,291,292,293,294,295,296,297,298,299,300,301,302,303,304,305,306,307,308,309,310,311,312,313,314,315,316,317,318,319,320,321,322,323,324,325,326,327,328,329,330,331,332,333,334,335,336,337,338,339,340,341,342,343,344,345,346,347,348,349,350,351,352,353,354,355,356,357,358,359,360,361,362,363,364,365,366,367,368,369,370,371,372,373,374,375,376,377,378,379,380,381,382,383,384,385,386,387,388,389,390,391] are cited in Supplementary Materials.
