# Supplementary material for: Identifying General Tumor and Specific Lung Cancer Biomarkers by Transcriptomic Analysis
Source: Biology (Basel). 2022 Jul 20;11(7):1082. doi: 10.3390/biology11071082 (PMC9313083; doi:10.3390/biology11071082)
Supplement: Supplementary file 1 [file biology-11-01082-s001.zip › SP3 LC_OC.pdf]

Supplementary Table S3

Gene	Function	LC
**ASPM**	Involved in mitotic spindle regulation and coordination of mitotic processes. Regulating microtubule dynamics at spindle poles including spindle orientation, astral microtubule density and poleward microtubule flux seems to depend on the association with the katanin complex formed by KATNA1 and KATNB1. Enhances the microtubule lattice severing activity of KATNA1 by recruiting the katanin complex to microtubules. Can block microtubule minus-end growth and reversely this function can be enhanced by the katanin complex.	ASPM is involved in the malignant progression of gliomas, possibly through expansion of a cáncer stem cell compartment. shRNA silencing of ASPM resulted in dramatic proliferation arrest and cell death in two different gliomasphere models [125].
**AURKA**	Mitotic serine/threonine kinase that contributes to the regulation of cell cycle progression. Associates with the centrosome and the spindle microtubules during mitosis and plays a critical role in various mitotic events including the establishment of mitotic spindle, centrosome duplication, centrosome separation as well as maturation, chromosomal alignment, spindle assembly checkpoint, and cytokinesis. Required for normal spindle positioning during mitosis and for the localization of NUMA1 and DCTN1 to the cell cortex during metaphase. Required for initial activation of CDK1 at centrosomes. Phosphorylates numerous target proteins, including ARHGEF2, BORA, BRCA1, CDC25B, DLGP5, HDAC6, KIF2A, LATS2, NDEL1, PARD3, PPP1R2, PLK1, RASSF1, TACC3, p53/TP53 and TPX2. Regulates KIF2A tubulin depolymerase activity. Required for normal axon formation. Plays a role in microtubule remodeling during neurite extension. Important for microtubule formation and/or stabilization. Also acts as a key regulatory component of the p53/TP53 pathway, and particularly the checkpoint-response pathways critical for oncogenic transformation of cells, by phosphorylating and stabilizing p53/TP53. Phosphorylates its own inhibitors, the protein phosphatase type 1 (PP1) isoforms, to inhibit their activity. Necessary for proper cilia disassembly prior to mitosis.	AURKA expression was significantly up-modulated in tumor samples compared to matched lung tissue ($p<0.01$, mean log2(FC)=1.5). Moreover, AURKA was principally up-modulated in moderately and poorly differentiated lung cáncers ($p<0.01$), as well as in squamous and adenocarcinomas compared to the non-invasive bronchioloalveolar histotype (p=0.029). No correlation with survival was observed [90]. AURKA mRNA expression [hazard ratio (HR), 1.81; 95% confidence interval (CI), 1.16–2.84; P=0.009], age (HR, 1.03; 95% CI, 1.00–1.06; P=0.020), pathological tumor stage 2 (HR, 2.43; 95% CI, 1.16–5.10; P=0.019) and involvement of distal nodes (pathological node stage 2) (HR, 3.14; 95% CI, 1.24–7.99; P=0.016) were independent predictors of poor prognosis in patients with NSCLC [126]. Nongenetic resistance through the activation of AURKA by its coactivator **TPX2** emerges in response to chronic EGFR inhibition where it mitigates drug-induced apoptosis. Aurora kinase inhibitors suppress this adaptive survival program, increasing the magnitude and duration of EGFR inhibitor response in preclinical models. Treatment-induced activation of AURKA is associated with resistance to EGFR inhibitors in vitro, in vivo and in most individuals with EGFR-mutant lung adenocarcinoma [127]. The hazard ratio (HR) of the mRNA expression of AURKA for OS was 1.588 with (1.127-2.237) 95% confidence interval (CI) (P=0.009). The mRNA levels of CDC20 (HR 1.530, 95% CI 1.086-2.115, P=0.016) and TPX2 (HR 1.777, 95%CI 1.262-2.503, P=0.001) were also significantly associated with the OS. Expression of these three genes were not associated with RFS, suggesting that there might be many factors affecting recurrence-free survival [128]. Loss-of-function mutations in the retinoblastoma gene RB1 are common in several treatment-refractory cáncers such as small-cell lung cáncer and triple negative breast cáncer. The identification of a synthetic lethal interaction between RB1 and AURKA inhibition, and the discovery of a drug that can be dosed continuously to achieve uninterrupted inhibition of AURKA kinase activity without myelosuppression, suggest a new approach for the treatment of RB1-deficient malignancies, including patients progressing on CDK4/6 inhibitors [129]. MT477 inhibited the kinase AURKA (Aurora A) by 77±1%. This member of the serine/threonine kinases is strongly implicated in mitosis and meiosis and its activity is responsible for healthy cell proliferation [130]. It regulates cell-cycle events from late S-phase through M-phase, including centrosome maturation, mitotic entry, centrosome separation and bipolar spindle assembly [131]. KRAS positively regulates AURKA and AURKB expression. Furthermore, in KRAS-positive H358 and A549 cell lines, inducible knockdown of AURKA or AURKB, as well as treatment with a dual AURKA/AURKB inhibitor, decreased growth, viability, proliferation, transformation, and induced apoptosis in vitro. In addition, inducible shRNA-mediated knockdown of AURKA in A549 cells decreased tumor growth in vivo. More importantly, dual pharmacological inhibition of AURKA and AURKB reduced growth, viability, transformation, and induced apoptosis in vitro in an oncogenic KRAS-dependent manner, indicating that Aurora kinase

		inhibition therapy can specifically target KRAS-transformed cells [91].
**AURKB**	Serine/threonine-protein kinase component of the chromosomal passenger complex (**CPC**), a key regulator of mitosis, in the centromere ensure correct chromosome alignment and segregation and is required for chromatin-induced microtubule stabilization and spindle assembly. Key component of the cytokinesis checkpoint, a process required to delay abscission to prevent both premature resolution of intercellular chromosome bridges and accumulation of DNA damage.	AURKB activation is associated with acquired resistance to EGFR tyrosine kinase inhibitors (EGFR TKIs), and AURKB constitutes a potential target in NSCLC progressing to anti-EGFR therapy and not carrying resistance mutations [132]. KRAS-positive H358 and A549 cell lines, inducible knockdown of AURKA or AURKB, as well as treatment with a dual AURKA/AURKB inhibitor, decreased growth, viability, proliferation, transformation, and induced apoptosis in vitro in an oncogenic KRAS-dependent manner, indicating that Aurora kinase inhibition therapy can specifically target KRAS-transformed cells [91].
**BIRC5**	Multitasking protein that has dual roles in promoting cell proliferation and preventing apoptosis. Component of a chromosome passage protein complex (**CPC**) which is essential for chromosome alignment and segregation during mitosis and cytokinesis. Regulator of the localization of this complex; directs CPC movement to different locations from the inner centromere during prometaphase to midbody during cytokinesis and participates in the organization of the center spindle by associating with polymerized microtubules. Involved in the recruitment of CPC to centromeres during early mitosis via association with histone H3 phosphorylated at 'Thr-3' (H3pT3) during mitosis. The complex with RAN plays a role in mitotic spindle formation by serving as a physical scaffold to help deliver the RAN effector molecule **TPX2** to microtubules. May counteract a default induction of apoptosis in G2/M phase. The acetylated form represses STAT3 transactivation of target gene promoters. Inhibitor of **CASP3** and **CASP7**. Isoform 2 and isoform 3 do not appear to play vital roles in mitosis. Isoform 3 shows a marked reduction in its anti-apoptotic effects when compared with the displayed wild-type isoform.	For TN adenocarcinomas, BIRC5 may serve as a promising prognostic predictor and therapeutic target. The upregulation of BIRC5 may enable the activation of the cell cycle signaling pathway to participate in the development of this specific type of LAD [233] [105]. A history of smoking is associated with increased nuclear **survivin** and Ki-67 expression in lung adenocarcinomas of p-stage I, but not p-stages II or III. In smokers, the nuclear survivin and Ki-67 expression in p-stage I adenocarcinomas was lower than that of other p-stage I lung cáncer types, and was associated with an enhanced survival rate [133]. Downregulation of survivin expression dramatically decreased the invasive and metastatic capacities of the cells, suppressed the proliferation, decelerated the rate of growth, reduced the number of clones on soft agar and decreased the capacity of RBM penetration and migration [134]. Survivin overexpression is almost always present in early-stage NSCLC, suggesting that this protein may play a role in lung tumorigenesis [135]. .
**BUB1**	Serine/threonine-protein kinase that performs 2 crucial functions during mitosis: it is essential for spindle-assembly checkpoint signaling and for correct chromosome alignment. Has a key role in the assembly of checkpoint proteins at the kinetochore, being required for the subsequent localization of CENPF, BUB1B, CENPE and MAD2L1. Required for the kinetochore localization of PLK1. Required for centromeric enrichment of AUKRB in prometaphase.	BUB gene family members including BUB1, BUBR1 and BUB3 are not frequent targets for mitotic checkpoint defects in lung cáncers, if present at all [136].
**BUB1B**	Kinase involved in spindle-assembly checkpoint function acting as an inhibitor of the APC/C, facilitating catalysis at the kinetochore and chromosome alignment during metaphase segregation [137]. Essential component of the mitotic checkpoint. Required for normal mitosis progression.	BUB1B regulates anchorage-independent growth, anoikis and metastasis, and its overexpression plays a critical role in poor prognosis and tumor progression of lung adenocarcinoma [138].
**CCNA2**	A highly conserved cyclin family and is expressed in almost all tissues in the human body, related to the control of cell cycle at the G1/S and the G2/M transitions [139]. Functions through the formation of specific serine/threonine protein kinase holoenzyme complexes with the cyclin-dependent protein kinases CDK1 or CDK2. The cyclin subunit confers the substrate specificity of these complexes and differentially interacts with and activates CDK1 and CDK2 throughout the cell cycle.	CCNA2 is overexpressed in dozens of cáncer types, suggesting a potential role in cáncer transformation and progression, and It might be involved in the processes of epithelial-mesenchymal transitions (EMT) and metastasis [140]. CCNA2 facilitates epithelial-to-mesenchymal transition via the integrin $\alpha v\beta 3$ signaling in NSCLC [141].
**CCNB1**	Essential for the control of the cell cycle at the G2/M (mitosis) transition.	Cyclin B1 may be dysregulated in NSCLC, particularly in the SCC subtype, and that a high level of cyclin B1 expression may be a prognostic marker for patients with early-stage SCC of the lung [142]. A higher level of CCNB1 indicates a larger tumor size and a higher probability of metastasis. CCNB1 expression can act as a prognostic predictor [143]. CDK1-CCNB1 complexes can induce abnormal regulation of downstream protein phosphorylation, leading to uncontrolled cell proliferation [144]. Selective inhibition of CCNB1 or CDKs can limit the progress of the cell cycle and/or induce cellular apoptosis. CCNB1 or CDK1 inhibition could suppress the occurrence and development of tumors, via the p53/Bax apoptosis pathway in tumor cells [145].
**CCNB2**	CCNB1 and 2 are cyclins that form the complex maturation promoting factor (MPF) with p34, a cyclin 1-dependent kinase (Cdk1), to control	CCNB2 increased 5.468-fold (P = 0.0005) in IPAH, and 1.974-fold (P = 0.009) in HPAH [402]. The upregulation of FOXM1 and its downstream targets **CCNB1,**

	mitosis [146,147].	**CCNB2 and TOP2A** is involved in sepsis-related acute respiratory distress syndrome [403]. High levels of CCNB2 protein are positively associated with the status of differentiated degree, tumor size, lymph node metastasis, distant metastasis, and clinical stage [148].
**CDC20**	Required for full ubiquitin ligase activity of the anaphase promoting complex/cyclosome (APC/C) and may confer substrate specificity upon the complex. Is regulated by MAD2L1: in metaphase the MAD2L1-CDC20-APC/C ternary complex is inactive and in anaphase the CDC20-APC/C binary complex is active in degrading substrates. The CDC20-APC/C complex positively regulates the formation of synaptic vesicle clustering at the active zone to the presynaptic membrane in postmitotic neurons. CDC20-APC/C-induced degradation of NEUROD2 induces presynaptic differentiation.	A relatively higher expression of Cdc20 was associated with pleural invasion, and shorter 5-year overall survival in NSCLC in a gender-specific manner (male specificity) [149]. In line with this notion, deregulation of Cdc20 was observed in lung adenocarcinoma tissue samples [150]. Notably, knockdown of Cdc20 inhibited cell growth, induced G2/M cell cycle arrest, and retarded colony formation of lung cáncer cells [151]. In lung adenocarcinoma patients, overexpression of cell division cycle 20 was significantly associated with bigger primary tumor size ($p = 0.0023$), higher MKI67 level ($r = 0.7618$, $p < 0.0001$), higher DNA ploidy level ($p < 0.0001$), and poor prognosis (hazard ratio = 2.39, confidence interval: 1.87–3.05, $p < 0.0001$) [152].
**CDC6**	Involved in the initiation of DNA replication. Also participates in checkpoint controls that ensure DNA replication is completed before mitosis is initiated.	miR26a and miR26b inhibit replication licensing and the proliferation, migration, and invasion of lung cáncer cells by targeting CDC6 [92]. For characterization of malignant, metaplastic or dysplastic cells, CDC6 protein had the highest sensitivity of 87.7%. All the three markers (MCM2, MCM5, CDC6) together had a sensitivity of 94.4%. Furthermore, these proteins could be employed to assess the proliferative potential of precáncerous or atypical cells, as overexpression increases with the stage of disease and degree of metastasis [153]. Pharmacological and genetic studies demonstrated CDC6 is positively controlled by mTORC1-S6K1 and mTORC2 signaling. miRNA screening revealed mTOR signaling suppresses miR-3178 thereby upregulating CDC6. Analysis of TCGA data found that CDC6 is overexpressed in most cáncers and associated with the poor survival of cáncer patients. Our findings suggest that mTOR signaling may control DNA replication origin licensing and replisome stability thereby cell cycle progression through CDC6 regulation [154]. Cdc6 transcription is regulated by the E2F transcription factors [93,94]. a strong correlation between increased Cdc6 expression and reduced E-cadherin levels [95]. The expression of five genes, that is, POLQ, PLK1, RAD51, CLASPIN and CDC6 was associated with overall, disease-free and relapse-free survival. This 'replication stress' metamarker may be a reliable predictor of survival for NSCLC, and may also help understand the molecular mechanisms underlying tumor progression [155]. eight candidate genes (DLGAP5, KIF11, RAD51AP1, CCNB1, AURKA, CDC6, OIP5 and NCAPG) are closely related to survival in LUAD. Low abundance of E6-associated protein (E6AP) promotes lung tumorigenesis by silencing p16 in a methylation-independent manner: in the context of low or lost expression of E6AP, E2F transcription factor 1 (E2F1) is free to stimulates cell division control protein 6 (CDC6) expression, which represses the INK4/ARF locus and contributes to p16 loss [156]. Ang- (1-7) inhibits lung cáncer cell growth by interrupting pre-replicative complex assembly and restrains epithelial-mesenchymal transition via Cdc6 inhibition. Furthermore, we constructed a mutant adeno-associated viral vector AAV8 (Y733F) that produced stable and highly efficient Ang-(1-7) expression in a xenograft tumor model. The results show that AAV8-mediated Ang-(1-7) over-expression can remarkably suppress tumor growth in vivo by down-regulating Cdc6 and anti-angiogenesis. Ang-(1-7) over-expression via the AAV8 method may be a promising strategy for lung cáncer treatment [96].
**CENPE**	Microtubule plus-end-directed kinetochore motor which plays an important role in chromosome congression, microtubule-kinetochore conjugation and spindle assembly checkpoint activation. Drives chromosome congression (alignment of chromosomes at the spindle equator resulting in the formation of the metaphase plate) by mediating the lateral sliding of polar chromosomes along spindle microtubules towards	CENPE can promote the proliferation of LUAD cells and is directly regulated by FOXM1 [404]. CENP-E is thus essential for the maintenance of chromosomal stability through efficient stabilization of microtubule capture at kinetochores [157].

	the spindle equator and by aiding the establishment and maintenance of connections between kinetochores and spindle microtubules.	Loss of the evolutionarily conserved gene enhancer of rudimentary homolog (ERH) leads to loss of CENP-E and consequently, chromosome congression defects and KRAS mutant cáncer cells are dependent on ERH for their survival [158].
**CENPF**	Required for kinetochore function and chromosome segregation in mitosis. Required for kinetochore localization of dynein, LIS1, NDE1 and NDEL1. Regulates recycling of the plasma membrane by acting as a link between recycling vesicles and the microtubule network through its association with STX4 and SNAP25. Acts as a potential inhibitor of pocket protein-mediated cellular processes during development by regulating the activity of RB proteins during cell division and proliferation. May play a regulatory or permissive role in the normal embryonic cardiomyocyte cell cycle and in promoting continued mitosis in transformed, abnormally dividing neonatal cardiomyocytes. Interaction with RB directs embryonic stem cells toward a cardiac lineage.	The hub genes, including AURKB, BUB1B, KIF2C, HMMR, CENPF, and CENPU, were overexpressed compared with the normal group by Gene Expression Profiling Interactive Analysis, and associated with reduced overall survival in lung cáncer patients [159]. By combining functional pathway and protein-protein interaction (PPI) analyses, five hub genes were selected, including cell division cycle 20 (CDC20), centromere protein F (CENPF), kinesin family member 2C (KIF2C), BUB1 mitotic checkpoint serine/threonine kinase (BUB1) and ZW10 interacting kinetochore protein (ZWINT). After verifying that the mRNA level of these hub genes was also upregulated in NSCLC tissues by using the GSE10072 dataset and in cell lines by reverse transcription-quantitative polymerase chain reaction. [160].
**EXO1**	5'->3' double-stranded DNA exonuclease which may also possess a cryptic 3'->5' double-stranded DNA exonuclease activity. Functions in DNA mismatch repair (MMR) to excise mismatch-containing DNA tracts directed by strand breaks located either 5' or 3' to the mismatch. Also exhibits endonuclease activity against 5'-overhanging flap structures similar to those generated by displacement synthesis when DNA polymerase encounters the 5'-end of a downstream Okazaki fragment. Required for somatic hypermutation (SHM) and class switch recombination (CSR) of immunoglobulin genes. Essential for male and female meiosis.	An allele Exo1 K589E confers a significantly increased risk of lung cáncer. The Exo1 K589E AG and AA genotype in association with smoking conferred an increased risk of 1.7208 (95% confidence interval = 1.2188-2.4295) for lung cáncer [161]. EXO1 Glu589Lys polymorphism and its surrounding regions might be genetic susceptibility markers for lung cáncer in this study population [162]. An allele of EXO1 rs1047840 may confer modulating effects on the risk of lung cáncer and could be used as a marker for early detection and primary prevention [163]. Exo1 K589E Lys allele may be used as a novel biomarker for cáncer susceptibility, particularly in smokers [164].
**FANCI**	Plays an essential role in the repair of DNA double-strand breaks by homologous recombination and in the repair of interstrand DNA cross-links (ICLs) by promoting FANCD2 monoubiquitination by FANCL and participating in recruitment to DNA repair sites. Required for maintenance of chromosomal stability. Specifically binds branched DNA: binds both single-stranded DNA (ssDNA) and double-stranded DNA (dsDNA). Participates in S phase and G2 phase checkpoint activation upon DNA damage.	Genetic polymorphism of the FA genes is associated with inter-individual susceptibility to lung adenocarcinoma [165]. The FA complex I functions to activate FANCD2 and FANCI by mono-ubiquitinating the protein following response to DNA damage [166]. The activated FANCD2 and FANCI proteins are subsequently transported to subnuclear foci, which are thought to be the sites of DNA repair and also contain BRCA1, FANCD1/BRCA2, proliferating cell nuclear antigen (PCNA) and Rad51 [167,168].
**HMMR**	Receptor for hyaluronic acid (HA). Involved in cell motility. When hyaluronan binds to HMMR, the phosphorylation of a number of proteins, including PTK2/FAK1 occurs. May also be involved in cellular transformation and metastasis formation, and in regulating extracellular-regulated kinase (ERK) activity. May act as a regulator of adipogenesis.	Knockdown of HMMR in LUAD cells decreased their potential to invade, migrate, and form colonies in vitro [169,170].
**KIF11**	Motor protein that crisscrosses antiparallel microtubules in the spindle [171]. KIF11 is part of the mitosis-associated EXATH protein complex that contributes to mitotic spindle formation and maturation [172], and controls the correct re-arrangement of microtubules to prevent their degradation [173]. Motor protein required for establishing a bipolar spindle during mitosis. Required in non-mitotic cells for transport of secretory proteins from the Golgi complex to the cell surface.	KIF11 overexpression in NSCLC has been associated with poor patient survival [173], and tumor growth [174]. KIF11 is a potential therapeutic target in malignant pleural mesothelioma, a form of aggressive cáncer associated with exposure to asbestos, where it has been over-expressed, opening the possibility of developing kinesin-based drugs for the treatment [175].
**KIF23**	Component of the centralspindlin complex that serves as a microtubule-dependent and Rho-mediated signaling required for the myosin contractile ring formation during the cell cycle cytokinesis. Essential for cytokinesis in Rho-mediated signaling. Required for the localization of ECT2 to the central spindle. Plus-end-directed motor enzyme that moves antiparallel microtubules in vitro.	KIF23 transcript was found to be overexpressed in the great majority of metastatic lymph nodes from advanced lung cáncers and primary lung tumors. Inhibiting KIF23 expression effectively suppressed lung cáncer cell growth [176]. The elevated level of KIF23 could be due to an additional copy of chromosome 15 demonstrated in 90% of NSCLC cases [177].
**KIF2C**	In complex with KIF18B, it constitutes the major microtubule plus-end depolymerizing activity in mitotic cells. Regulates the turnover of microtubules at the kinetochore and functions in chromosome segregation during mitosis. Plays a role in chromosome congression and is required for the lateral to end-on conversion of the chromosome-microtubule attachment.	A Hub gene overexpressed in cáncer [159,160,178]. KIF2C was identified in the association with progression and prognosis of LUAD, which might refer to a poor prognosis probably by regulating the cell cycle signaling pathway [179]. The abnormal expression of KIF2C is associated with abnormal mitosis, chromosomal

		aberrations, and malignant transformation. Therefore, the deregulation of KIF2C expression can contribute to cáncer development and progression [159].
**MAD2L1**	Component of the spindle-assembly checkpoint that prevents the onset of anaphase until all chromosomes are properly aligned at the metaphase plate. Required for the execution of the mitotic checkpoint which monitors the process of kinetochore-spindle attachment and inhibits the activity of the anaphase promoting complex by sequestering CDC20 until all chromosomes are aligned at the metaphase plate.	Cyclin-dependent kinase 1 (CDK1) and MAD2 mitotic arrest deficient-like 1 (MAD2L1), two critical mitotic checkpoint genes, were selected for further study. Elevated expression of CDK1 and MAD2L1 was validated in an independent LUAD cohort. Kaplan-Meier analysis revealed that CDK1 and MAD2L1 expression was negatively correlated with both overall survival (OS) and relapse-free survival (RFS). In conclusion, CDK1 and MAD2L1 were adverse prognostic biomarkers for LUAD whose increased expression could render patients with LUAD a high risk of cáncer recurrence and poor survival, suggesting that they might be applied as potential targets for LUAD treatment [180]. MAD2L1 Leu84Met SNP was associated with increased risk of lung cáncer in an allele dose dependent manner, with the ORs being 2.55 (95% CI 1.95 to 3.33) for the Leu/Met and 2.68 (95% CI 2.05 to 3.48) for the Met/Met genotype compared with the Leu/Leu genotype [97]. GDP-mannose -4,6-dehydratase (GMDS) knockdown induced the expression of CDKN1A, CASP8, MAP3K7 and FAS while inhibited JUN, DDIT3, VEGFA, SKA1 and MAD2L1 at mRNA level. GMDS expression is significantly upregulated in lung adenocarcinoma at both mRNA and protein levels. Lentivirus-mediated shRNA strategy inhibited GMDS expression efficiently in human lung adenocarcinoma cells A549 and H1299, and GMDS knockdown impaired cell proliferation, colony formation ability, induced cell cycle arrest, and apoptosis in both cell lines [181].
**MCM2**	MCM2,4 and 7 are nuclear proteins for maintenance of mini-chromosomes that play an important role in cell division and DNA replication [182], are part of the pre-replication complex, form complexes between MCMs, and are regulated by proteins. kinases [183]. Acts as component of the MCM2-7 complex (MCM complex) which is the putative replicative helicase essential for 'once per cell cycle' DNA replication initiation and elongation in eukaryotic cells.	The deregulation of MCM2 is involved in lung cáncer cell proliferation, the cell cycle, and migration [184]. Prognostic significance of MCM2, Ki-67 and gelsolin in non-small cell lung cáncer [185]. MCM2 is detectable in 2-3 times more proliferating premalignant lung cells than is Ki-67. The promise of MCM2 as a sensitive marker for premalignant lung cells is enhanced by the fact that it is present in cells at the surface of metaplastic lung lesions, which are more likely to be exfoliated into sputum [186].
**MCM4**	Acts as component of the MCM2-7 complex (MCM complex) which is the putative replicative helicase essential for 'once per cell cycle' DNA replication initiation and elongation in eukaryotic cells. The active ATPase sites in the MCM2-7 ring are formed through the interaction surfaces of two neighboring subunits such that a critical structure of a conserved arginine finger motif is provided in trans relative to the ATP-binding site of the Walker A box of the adjacent subunit.	MCM4 may play an essential role in the proliferation of some NSCLC cells. High level of MCM4 expression has been associated with cyclin E expression in non–small cell lung carcinoma [187]. Patients with high MCM4 expression level (>70%) had significantly shorter survival in the adenocarcinoma group [188].
**MKI67**	Required to maintain individual mitotic chromosomes dispersed in the cytoplasm following nuclear envelope disassembly. Associates with the surface of the mitotic chromosome, the perichromosomal layer, and covers a substantial fraction of the chromosome surface. Prevents chromosomes from collapsing into a single chromatin mass by forming a steric and electrostatic charge barrier: the protein has a high net electrical charge and acts as a surfactant, dispersing chromosomes and enabling independent chromosome motility.	Proliferation index (overall mean: 40.7%) differed significantly according to histologic subtypes with SQCC showing a mean PI (52.8%) twice as high as ADC (25.8%) [189].
**MYBL2**	Transcription factor involved in the regulation of cell survival, proliferation, and differentiation. Transactivates the expression of the CLU gene.	MYBL2 is expressed in basically all proliferating cells [102]. MYBL2–MuvB complex is needed to increase target specificity for FOXM1 binding [190]. An adequate proliferative capacity mediated by MYBL2 is necessary to maintain genomic stability [103]. MYBL2 has also been shown to drive cell proliferation and/or cell cycle progression in cáncer cells, such as lung adenocarcinoma [104]. B-Myb Mediates Proliferation and Migration of Non-Small-Cell Lung Cáncer via Suppressing IGFBP3 [191]. Belongs to TF modules that were up-regulated in LUAD [192].
**NDC80**	Acts as a component of the essential kinetochore-associated NDC80 complex, which is required for chromosome segregation and spindle checkpoint activity. Required for kinetochore integrity and the organization of stable microtubule binding sites in the outer plate of the kinetochores. The NDC80 complex synergistically enhances the affinity of the SKA1 complex for microtubules and may allow the NDC80 complex to track depolymerizing microtubules. Plays a role in chromosome congression and is essential for the end-on attachment of the	TAI-1 showed strong potency across a broad spectrum of tumor cells. TAI-1 disrupted Hec1-Nek2 protein interaction, led to Nek2 degradation, induced significant chromosomal misalignment in metaphase, and induced apoptotic cell death [98]. overexpression of Hec1 in an inducible mouse model results in mitotic

	kinetochores to spindle microtubules.	checkpoint hyperactivation. As previously observed with overexpression of the Mad2 gene, hyperactivation of the mitotic checkpoint leads to aneuploidy in vitro and is sufficient to generate tumors in vivo that harbor significant levels of aneuploidy. These results underscore the role of chromosomal instability as a result of mitotic checkpoint hyperactivation in the initiation of tumorigenesis [193].
**NEK2**	Protein kinase which is involved in the control of centrosome separation and bipolar spindle formation in mitotic cells and chromatin condensation in meiotic cells. Regulates centrosome separation (essential for the formation of bipolar spindles and high-fidelity chromosome separation) by phosphorylating centrosomal proteins such as CROCC, CEP250 and NINL, resulting in their displacement from the centrosomes. Regulates kinetochore microtubule attachment stability in mitosis via phosphorylation of NDC80. Involved in regulation of mitotic checkpoint protein complex via phosphorylation of CDC20 and MAD2L1. Plays an active role in chromatin condensation during the first meiotic division through phosphorylation of HMGA2. Phosphorylates: PPP1CC; SGO1; NECAB3 and NPM1. Essential for localization of MAD2L1 to kinetochore and MAPK1 and NPM1 to the centrosome. Phosphorylates CEP68 and CNTLN directly or indirectly. NEK2-mediated phosphorylation of CEP68 promotes CEP68 dissociation from the centrosome and its degradation at the onset of mitosis. Involved in the regulation of centrosome disjunction.	Overexpression of NEK2 significantly increased cell proliferation in normal fibroblasts and in cell lines of lung cáncer [194,195]. NEK2 may be a more effective tumor proliferation marker of poor prognosis for NSCLC patients, and that NEK2 may represent a novel potential target for NSCLC therapeutic intervention [196]. NEK2 is involved in the phosphorylation of β-catenin at Ser33/Ser37. Treatment with CGK062, a small chemical molecule, which promotes the phosphorylation of β-catenin at Ser33/Ser37 through protein kinase C (PKC)α to induce its degradation, reduced β-catenin levels and inhibited the CUG2-induced features of malignant tumors, including increased cell migration, invasion and sphere formation [197]. NEK2 over-expression induced chromosomal instability, promoted cell proliferation, down-regulated pro-apoptotic PUMA and BAD, up-regulated anti-apoptotic BCL-2 and MCL-1, increased phosphor-AKT and phosphor-S6 levels, and enhanced nuclear accumulation of β-catenin [198]. A novel role for Nek2 in promoting metastasis in addition to its currently defined role in promoting chromosomal instability [199]. Among Nek2 kinase, MCM7, and Ki-67, it is Nek2 kinase that is the most effective tumor proliferation marker of poor prognosis for NSCLC patients [200].
**RFC4**	The elongation of primed DNA templates by DNA polymerase delta and epsilon requires the action of the accessory proteins proliferating cell nuclear antigen (PCNA) and activator 1. This subunit may be involved in the elongation of the multiprimed DNA template. RFC4 encodes the fourth largest subunit of the RFC complex, which helps PCNA load onto DNA in an ATP-dependent process during DNA synthesis and serves an important role in DNA repair activities following DNA damage.	The most significant 10 node degree genes were topoisomerase IIα (TOP2A), proliferating cell nuclear antigen (PCNA), replication factor C subunit 4 (RFC4), checkpoint kinase 1 (CHEK1), thymidylate synthase (TYMS), minichromosome maintenance protein (MCM) 2, cell division cycle (CDC) 20, cyclin dependent kinase inhibitor 3 (CDKN3), MCM3 and CDC6, which were also primarily associated with ‘cell cycle’, ‘DNA replication’ and ‘oocyte meiosis’ signaling pathways [201]. The DEGs in the lung squamous cell carcinoma group were significantly enriched in the following three pathways: Hsa04110, cell cycle (P=3.93E-06), involving genes YWAQ, MCM3, CHEK2, GADD45 G, RAD21 and PCNA; hsa03030, DNA replication (P=8.50E-06), involving genes MCM3, FEN1, RFC4, POLA1, PCNA and MCM2; and hsa03430, mismatch repair (P=0.01309), involving genes RFC4, MSH6, RFC2 and MSH3 [202]. CCNB2, PLK1, KIF2C, CENPA, CENPF, BUB1, BUB1B, BIRC5, CENPE, ZWINT, AURKB, CHEK1, EXO1, RAD51, and RFC4 can interact with each other and this interaction was predominantly associated with protein serine/threonine kinase activity [178]. QPCR analysis confirmed coordinate overexpression of four genes (COPB2,ELF3, RFC4, and PLS1) in an independent set of LAC samples [203].
**SPAG5**	Essential component of the mitotic spindle required for normal chromosome segregation and progression into anaphase. Required for chromosome alignment, normal timing of sister chromatid segregation, and maintenance of spindle pole architecture. In complex with SKAP, it promotes stable microtubule-kinetochore attachments. May contribute to the regulation of separase activity. May regulate AURKA localization to mitotic spindle, but not to centrosomes and CCNB1 localization to both mitotic spindle and centrosomes. Involved in centriole duplication. Required for CDK5RAP2, CEP152, WDR62 and CEP63 centrosomal localization and promotes the centrosomal localization of CDK2.	MicroRNA-1179 suppresses cell growth and invasion by targeting sperm-associated antigen 5-mediated Akt signaling in human non-small cell lung cáncer [204]. SPAG5 was upregulated in most of the lung adenocarcinoma cell lines as compared to normal lung epithelial cells. SPAG5 knockdown suppressed proliferation, colony forming, and migration of lung adenocarcinoma A549 cells in vitro and inhibited tumor growth in vivo. p53 suppression is essential for oncogenic SPAG5 upregulation in lung adenocarcinoma [205]. We identified novel genes whose expression was upregulated in NSCLC, including SPAG5, POLH, KIF23, and RAD54L, which are associated with mitotic spindle formation, DNA repair, chromosome segregation, and dsDNA break repair, respectively [206].

**TIMEL**	Plays an important role in the control of DNA replication, maintenance of replication fork stability, maintenance of genome stability throughout normal DNA replication, DNA repair and in the regulation of the circadian clock. Required to stabilize replication forks during DNA replication by forming a complex with TIPIN: this complex regulates DNA replication processes under both normal and stress conditions, stabilizes replication forks and influences both CHEK1 phosphorylation and the intra-S phase checkpoint in response to genotoxic stress.	TIMELESS (TIM) is a mammalian homolog of a Drosophila circadian rhythm gene, but its circadian properties in mammals have yet to be determined. TIM appears to be essential for replication protection and genomic stability. TIM knockdown suppressed proliferation and clonogenic growth, and induced apoptosis in H157 and H460 cells [99].
**TOP2A**	Control of topological states of DNA by transient breakage and subsequent rejoining of DNA strands. Topoisomerase II makes double-strand breaks. Essential during mitosis and meiosis for proper segregation of daughter chromosomes. May play a role in regulating the period length of RNATL/BMAL1 transcriptional oscillation. This gene encodes a DNA topoisomerase, an enzyme that controls and alters the topologic states of DNA during transcription. This nuclear enzyme is involved in processes such as chromosome condensation, chromatid separation, and the relief of torsional stress that occurs during DNA transcription and replication.	High expression of two topoisomerase isoforms, TOP2A and TOP3A, was found to be correlated to worse overall survival (OS) in all NSCLC and lung adenocarcinoma (Ade) patients, but not in lung squamous cell carcinoma (SCC) patients [207,208]. Topoisomerase II alpha (TOP2A) is another proliferation marker that has potential to be used to distinguish bronchopulmonary neuroendocrine neoplasms (BP-NEN) entities. [209].
**TPX2**	Spindle assembly factor required for normal assembly of mitotic spindles. Required for normal assembly of microtubules during apoptosis. Required for chromatin and/or kinetochore dependent microtubule nucleation. Mediates AURKA localization to spindle microtubules. Activates AURKA by promoting its autophosphorylation at 'Thr-288' and protects this residue against dephosphorylation. TPX2 is inactivated upon binding to importin-alpha. At the onset of mitosis, GOLGA2 interacts with importin-alpha, liberating TPX2 from importin-alpha, allowing TPX2 to activate AURKA kinase and stimulate local microtubule nucleation.	TPX2, KIF11 and CKAP5 are highly overexpressed in NSCLC [173]. Elevated mRNA Levels of AURKA, CDC20 and TPX2 are associated with poor prognosis of smoking-related lung adenocarcinoma [128]. Depletion of TPX2 or its associated kinase Aurora-A preferentially reduced cell viability in a panel of BRCA2-deficient cáncer cells. RCA2-deficient cáncer cells show enhanced sensitivity to inactivation of TPX2 or its partner Aurora-A, which points at an actionable dependency of genomically unstable cáncers [210].
**TTK**	Phosphorylates proteins on serine, threonine, and tyrosine. Probably associated with cell proliferation. Essential for chromosome alignment by enhancing AURKB activity (via direct CDCA8 phosphorylation) at the centromere, and for the mitotic checkpoint.	LMO1 functions as an oncogene by regulating TTK expression and correlates with neuroendocrine differentiation of lung cáncer [211]. USP9X stabilized TTK via direct interaction and efficient deubiquitination of TTK on K48 ubiquitin chain. Moreover, knockdown of USP9X or TTK inhibited cell proliferation, migration and tumorigenesis, and the immunohistochemical analysis of clinical NSCLC samples showed that the protein expression levels of USP9X and TTK were significantly elevated and positively correlated in tumor tissues [212]. TTK antagonism is hypothesized to cause genomic instability and cell death.CFI-402257 is a highly selective TTK inhibitor. CFI-402257 triggered aneuploidy and apoptotic death of lung cáncer cells without changing centrosome number [213]. Expression of TTK, a Novel Human Protein Kinase, Is Associated with Cell Proliferation [214].
**ZWINT**	Part of the MIS12 complex, which is required for kinetochore formation and spindle checkpoint activity. Required to target ZW10 to the kinetochore at prometaphase.	Knockdown of ZWINT reduced proliferation in NCI H226 and A549 cells ($P < 0.05$). Knockdown also inhibited cell migration, invasion, apoptosis, and colony formation ($P < 0.05$). ZWINT knockdown reduced tumor volume ($P < 0.05$). KEGG analysis revealed that TNF, P53, and PI3K signal networks would be the most potential ZWINT-related pathways and were identified by Western blot analysis [100]. disease stage and expression level of ZWINT were correlated with recurrence-free survival and OS in lung cáncer [215].

Alineamiento	Function	LC
**HEG1**	Receptor component of the CCM signaling pathway which is a crucial regulator of heart and vessel formation and integrity May act through the stabilization of endothelial cell junctions	Cyclin D1 (CCND1), Ajuba LIM Protein (AJUBA), and heart development protein with EGF like domains 1 (HEG1) were identified as common target genes of miR-193b-3p and -5p. A reporter assay and an Ago2-RIP experiment showed that both

		miRNAs directly bind to the 3' untranslated region (3'UTR) of the target mRNA. Knockdown of target genes reduced the proliferative and metastatic potential of primary and metastatic lung cáncer cells. Our results demonstrate miR-193b is a dual-strand tumor suppressor and a novel therapeutic target for lung cáncer [216].
**PLSCR4**	May mediate accelerated ATP-independent bidirectional transbilayer migration of phospholipids upon binding calcium ions that results in a loss of phospholipid asymmetry in the plasma membrane. May play a central role in the initiation of fibrin clot formation, in the activation of mast cells and in the recognition of apoptotic and injured cells by the reticuloendothelial system.	LINC00641 suppressed cell proliferation and induced cell apoptosis in NSCLC, indicating that LINC00641 exerted a tumor-suppressive role in NSCLC. Through mechanism investigation, we determined that LINC00641 acted as a competing endogenous RNA (ceRNA) in NSCLC by sponging miR-424-5p to upregulate phospholipid scramblase (PLSCR4) expression [217]. Peripheral Immune Cell Gene Expression Changes in Advanced Non-Small Cell Lung Cáncer Patients Treated with First Line Combination Chemotherapy [218].
**GMFG**	GMFG (Glia Maturation Factor Gamma) is a Protein Coding gene. Among its related pathways are GPCR Pathway and Nanog in Mammalian ESC Pluripotency. Gene Ontology (GO) annotations related to this gene include actin binding and enzyme activator activity. An important paralog of this gene is GMFB.	Genes Differentially Expressed (T versus NT) in Macro and LCM Samples of NSCLC, All Three Histologies Combined [219]. Genes downregulated in BRG1 knockdown cell lines [220].
**FCGR3B**	Receptor for the Fc region of immunoglobulins gamma. Low affinity receptor. Binds complex or aggregated IgG and also monomeric IgG. Contrary to III-A, it is not capable of mediating antibody-dependent cytotoxicity and phagocytosis. May serve as a trap for immune complexes in the peripheral circulation which does not activate neutrophils.	A higher burden of the CNVs was found in 10–50 kb length. The 16 CNV-driven genes mainly located in chr 1 and chr 3 were enriched in immune response [e.g. complement factor H (CFH) and Fc fragment of IgG, low affinity IIIa, receptor (FCGR3A)], starch and sucrose metabolism [e.g. amylase alpha 2A (AMY2A)]. Furthermore, 38 TFs were screened for the 9 CNV-driven genes and then the regulatory network was constructed, in which the GATA-binding factor 1, 2, and 3 (GATA1, GATA2, GATA3) jointly regulated the expression of TP63 [221].
**NME4**	Major role in the synthesis of núcleoside triphosphates other than ATP. The ATP gamma phosphate is transferred to the NDP beta phosphate via a ping-pong mechanism, using a phosphorylated active-site intermediate. Through the catalyzed exchange of gamma-phosphate between di- and triphosphonúcleosides participates in regulation of intracellular núcleotide homeostasis.	NME4 may enhance non-small cell lung cáncer progression by overcoming cell cycle arrest and promoting cellular proliferation [222].
**SNRK**	May play a role in hematopoietic cell proliferation or differentiation. Potential mediator of neuronal apoptosis.	SNRK/SP1-ITGB1 signaling axis promotes angiogenesis in vivo [223].
**BIRC5**	Multitasking protein that has dual roles in promoting cell proliferation and preventing apoptosis. Component of a chromosome passage protein complex (CPC) which is essential for chromosome alignment and segregation during mitosis and cytokinesis. Regulator of the localization of this complex; directs CPC movement to different locations from the inner centromere during prometaphase to midbody during cytokinesis and participates in the organization of the center spindle by associating with polymerized microtubules. Involved in the recruitment of CPC to centromeres during early mitosis via association with histone H3 phosphorylated at 'Thr-3' (H3pT3) during mitosis. The complex with RAN plays a role in mitotic spindle formation by serving as a physical scaffold to help deliver the RAN effector molecule TPX2 to microtubules. May counteract a default induction of apoptosis in G2/M phase. The acetylated form represses STAT3 transactivation of target gene promoters. Inhibitor of CASP3 and CASP7. Isoform 2 and isoform 3 do not appear to play vital roles in mitosis. Isoform 3 shows a marked reduction in its anti-apoptotic effects when compared with the displayed wild-type isoform.	For TN adenocarcinomas, BIRC5 may serve as a promising prognostic predictor and therapeutic target. The upregulation of BIRC5 may enable the activation of the cell cycle signaling pathway to participate in the development of this specific type of LAD [105]. A history of smoking is associated with increased nuclear **survivin** and Ki-67 expression in lung adenocarcinomas of p-stage I, but not p-stages II or III. In smokers, the nuclear survivin and Ki-67 expression in p-stage I adenocarcinomas was lower than that of other p-stage I lung cáncer types, and was associated with an enhanced survival rate [133]. Downregulation of survivin expression dramatically decreased the invasive and metastatic capacities of the cells, suppressed the proliferation, decelerated the rate of growth, reduced the number of clones on soft agar and decreased the capacity of RBM penetration and migration [134]. Survivin overexpression is almost always present in early-stage NSCLC, suggesting that this protein may play a role in lung tumorigenesis [135].
**GIMAP5**	Plays a role in T lymphocyte development and the optimal generation of CD4/CD8 double-positive thymocytes. Inhibitor of GSK3A, possibly by sequestering GSK3A in cytoplasmic vesicles and impairing its translocation to the nucleus. Consequently, impairs GSK3A-dependent transcriptional program and regulation of the DNA damage response occurring during T cells proliferation. Required for the survival of peripheral T cells, natural killer (NK) and NK T-cell development and the maintenance of normal liver function.	overexpression of GIMAPs was implicated in certain types of lung cáncer[115].

**HBB**	Depletion of HBB in CTC-derived cultures has minimal effects on primary tumor growth, but it greatly increases apoptosis following ROS exposure, and dramatically reduces CTC-derived lung metastases. These effects are reversed by the antioxidant N-Acetyl Cysteine. Conversely, overexpression of HBB is sufficient to suppress intracellular ROS within CTCs. Altogether, these observations suggest that β-globin is selectively deregulated in cáncer cells, mediating a cytoprotective effect during blood-borne metastasis.	Depletion of HBB in CTC-derived cultures has minimal effects on primary tumor growth, but it greatly increases apoptosis following ROS exposure, and dramatically reduces CTC-derived lung metastases. These effects are reversed by the antioxidant N-AcetylCysteine. Conversely, overexpression of HBB is sufficient to suppress intracellular ROS within CTCs. Altogether, these observations suggest that β-globin is selectively deregulated in cáncer cells, mediating a cytoprotective effect during blood-borne metastasis.[224].
**IL33**	Cytokine that binds to and signals through the IL1RL1/ST2 receptor which in turn activates NF-kappa-B and MAPK signaling pathways in target cells. Involved in the maturation of Th2 cells inducing the secretion of T-helper type 2-associated cytokines. Also involved in activation of mast cells, basophils, eosinophils and natural killer cells. Acts as a chemoattractant for Th2 cells, and may function as an "alarmin", that amplifies immune responses during tissue injury	The IL-33/ST2 axis is emerging as a powerful modulator of the tumor microenvironment (TME) by recruiting immune cells, able to modify the TME, supporting malignant proliferation or improving antitumor immunity [225]. IL-33 signaling fuels outgrowth and metastasis of human lung cáncer [226]. Circulating IL-33 level is associated with the progression of lung cáncer [227]. IL-33 blockade suppresses tumor growth of human lung cáncer through direct and indirect pathways in a preclinical model [228]. Serum IL-33 as a Diagnostic and Prognostic Marker in Non Small Cell Lung Cáncer [229].
**AKAP12**	Anchoring protein that mediates the subcellular compartmentation of protein kinase A (PKA) and protein kinase C (PKC).	AKAP12 alpha expression is assoCiated with DNA methylation of the promoter region in lung cáncer, and that AKAP12 alpha may play an important role in lung cáncer carcinogenesis [230]. Promoter Methylation of Genes in and around the Candidate Lung Cáncer Susceptibility Locus 6q23-25 [231]. The risk genotypes of rs7753153 and rs985192, two SNPs in the multivariate analysis model associated with lung adenocarcinoma risk, were associated with the decreased expression level of AKAP12. This finding may suggest the potential interaction between AKAP12 expression and ESR1 SNPs in lung carcinogenesis [232]. TFAP2C blocked AKAP12-mediated cyclin D1 inhibition by inducing the overexpression of oncogenic microRNA (miRNA)-183 and simultaneously activated cyclin-dependent kinase 6-mediated cell cycle progression by downregulating tumor-suppressive miRNA-33a [233].
**CD93**	Receptor (or element of a larger receptor complex) for C1q, mannose-binding lectin (MBL2) and pulmonary surfactant protein A (SPA). May mediate the enhancement of phagocytosis in monocytes and macrophages upon interaction with soluble defense collagens. May play a role in intercellular adhesion.	One strategy for cáncer therapy is to inhibit the development of blood vessels in the tumor. Researchers at IGP show in a new study how the protein CD93 interacts with the protein network that is required for tumor vessels to form properly. Blocking this interaction could be used as a means to hamper blood vessel development and slow down the cáncer [234].
