# Supplementary material for: Identifying General Tumor and Specific Lung Cancer Biomarkers by Transcriptomic Analysis
Source: Biology (Basel). 2022 Jul 20;11(7):1082. doi: 10.3390/biology11071082 (PMC9313083; doi:10.3390/biology11071082)
Supplement: Supplementary file 1 [file biology-11-01082-s001.zip › SP4 LCII.pdf]

Supplementary Table S4

Gene	Function	LC
**ABCA3**	Plays an important role in the formation of pulmonary surfactant, probably by transporting lipids such as cholesterol. Transport, ATP-binding, Núcleotide-binding.	ABCA3 Phenotype in Non-Small Cell Lung Cáncer Indicates Poor Outcome [235]. More than 200 ABCA3 mutations have been reported to date with approximately three quarters of patients presenting as compound heterozygotes. Recent advances in our understanding of the molecular basis underlying normal ABCA3 biosynthesis and processing, as well as the mechanisms of alveolar epithelial cell dysregulation caused by the expression of its mutant forms, are beginning to emerge [236]. The PI of ABCA3 and TTF-1 seems to be a suitable prognostic marker for NSCLC [237]. Variants of the ABCA3 gene might contribute to susceptibility to interstitial lung diseases in the Chinese population [238].
**ABCA9**	May play a role in monocyte differentiation and lipid homeostasis.	Prediction and verification of the influence of the rs367781716 SNP on the interaction of the TATA-binding protein with the promoter of the human ABCA9 gene [239]. ABCA9 is likely involved in monocyte differentiation and macrophage lipid homeostasis [240].
**ADCY4**	Catalyzes the formation of the signaling molecule cAMP in response to G-protein signaling.	Rs3181385 is a SNP located at miRNA binding site of ADCY4 gene, in the present study there is a bordering significant association with the risk of NSCLC [241]. Top hypermethylated and down-regulated genes in lung adenocarcinoma [242].
**ALDH3B1**	Oxidizes medium and long chain saturated and unsaturated aldehydes. Metabolizes also benzaldehyde. Low activity towards acetaldehyde and 3,4-dihydroxyphenylacetaldehyde. May not metabolize short chain aldehydes. Can use both NADP+ and NAD+ as electron acceptor. May have a protective role against the cytotoxicity induced by lipid peroxidation.	ALDH3B1 expression was upregulated in a high percentage of human tumors (lung > breast = ovarian > colon). Increased ALDH3B1 expression in tumor cells may confer a growth advantage or be the result of an induction mechanism mediated by increased oxidative stress [243].
**BMP5**	Induces cartilage and bone formation.	The mRNA level of BMP5 was significantly higher in lung adenocarcinoma tissues than that in lung squamous cell carcinoma tissues [244]. BMP5 expression is low in NSCLC, an epithelial type tumor [244]. p15/cyclin D1 pathway and BMP5 might contribute to PinX1-associated cell proliferation and cell cycle transition IN NSCLC [245].
**C1QTNF7**		Whole genome microarray analysis in non-small cell lung cáncer [246]. The 29 lung adenocarcinoma-associated methylation sites are located in 27 DEGs [247]. EGR1 target gene [248].
**C2orf40**	Probable hormone that may attenuate cell proliferation and induce senescence of oligodendrocyte and neural precursor cells in the central nervous system. ECRG4-induced senescence is characterized by G1 arrest, RB1 dephosphorylation and accelerated CCND1 and CCND3 proteasomal degradation. Protein: Augurin GeneECRG4.	Network Analysis of Lung Transcriptomics Reveals a Distinct B-Cell Signature in Emphysema [249].
**CBLC**	Acts as an E3 ubiquitin-protein ligase, which accepts ubiquitin from specific E2 ubiquitin-conjugating enzymes, and then transfers it to substrates promoting their degradation by the proteasome. Functionally coupled with the E2 ubiquitin-protein ligases UB2D1, UB2D2 and UB2D3. Regulator of EGFR mediated signal transduction; upon EGF activation, ubiquitinates EGFR. Isoform 1, but not isoform 2, inhibits EGF-stimulated MAPK1 activation. Promotes ubiquitination of SRC phosphorylated at 'Tyr-419'. I	Upregulation of E3 Ubiquitin Ligase CBLC Enhances EGFR Dysregulation and Signaling in Lung Adenocarcinoma [250]. Seven genes AKR1B10, CBLC, CYP24A1, ALDH3A1, S100P, PLUNC and LOC147166 were potential diagnostic markers for NSCLC when assessed in bioinformatic analyses of publicly available gene expression data [251].
**CDC25A**	Tyrosine protein phosphatase which functions as a dosage-dependent inducer of mitotic progression. Directly dephosphorylates CDK1 and stimulates its kinase activity. Also dephosphorylates CDK2 in complex with cyclin E, in vitro. Hydrolase, Protein phosphatase. Cell cycle, Cell division, Mitosis	One of the major causes for deregulation of Cdc25A transcription is dysfunction of some transcription repressors, particularly p53 [252]. mutation in p53 gene occurs frequently in a variety of cáncers, including lung cáncer [253]. Of the 10 known substrates of SCF (beta-TRCP) E3 ligases, the protein level of cell division cycle 25 (CDC25). A is clearly affected in these lung cáncer cells. Cells treated with CDC25A inhibitors become less invasive. Thus, loss of beta-TRCP1 may promote both growth and cell motility of lung cáncer cells, possibly through regulation of CDC25A and the MMP11 level [254]. overexpression of cdc25A and cdc25B is frequent and that it may play an important role in NSCLC [255]. a novel CDC25A transcript variant with codon 110 (Glutamine) deletion, that we termed CDC25AQ110del in NSCLC cells. In 9 (75%) of the 12 NSCLC cell lines, CDC25AQ110del expression accounted for more than 20% of the CDC25A transcripts. Biological effects of CDC25AQ110del were investigated in H1299 and HEK-293F cells using UV radiation, flow cytometry, cycloheximide treatment, and confocal microscopy.

		Compared to CDC25Awt, CDC25AQ110del protein had a longer half-life; cells expressing CDC25AQ110del were more resistant to UV irradiation and showed more mitotic activity [256]. CDC25A rs3731513 and rs1380053, CDC25C rs6861656, CDC25A haplotype T/A/A/A/C and CDC25C haplotype A/G/G/G/C were significantly associated with the NSCLC patients' progression-free survival [257].
**CDCA5**	Regulator of sister chromatid cohesion in mitosis stabilizing cohesin complex association with chromatin. May antagonize the action of WAPL which stimulates cohesin dissociation from chromatin. Cohesion ensures that chromosome partitioning is accurate in both meiotic and mitotic cells and plays an important role in DNA repair. Required for efficient DNA double-stranded break repair. Cell cycle, Cell division, Mitosis	Suppression of CDCA5 expression with siRNAs inhibited the growth of lung cáncer cells [258].
**CDCA7**	Participates in MYC-mediated cell transformation and apoptosis; induces anchorage-independent growth and clonogenicity in lymphoblastoid cells. Insufficient to induce tumorigenicity when overexpressed but contributes to MYC-mediated tumorigenesis. May play a role as transcriptional regulator. Apoptosis, Transcription, Transcription regulation	JPO1/CDCA7 expression is elevated in a significant fraction of human colon, rectum, ovary, lung, uterus and stomach cáncers [259]. CDCA7 was significantly overexpressed in LUAD compared to adjacent normal tissues. Furthermore, overexpression of CDCA7 was positively associated with more advanced clinical features. Silencing CDCA7 inhibited cell proliferation in LUAD through G1 phase arrest and induction of apoptosis [260].
**CHEK2**	Serine/threonine-protein kinase which is required for checkpoint-mediated cell cycle arrest, activation of DNA repair and apoptosis in response to the presence of DNA double-strand breaks. May also negatively regulate cell cycle progression during unperturbed cell cycles. Following activation, phosphorylates numerous effectors preferentially at the consensus sequence [L-X-R-X-X-S/T]. Regulates cell cycle checkpoint arrest through phosphorylation of CDC25A, CDC25B and CDC25C, inhibiting their activity. Inhibition of CDC25 phosphatase activity leads to increased inhibitory tyrosine phosphorylation of CDK-cyclin complexes and blocks cell cycle progression. May also phosphorylate NEK6 which is involved in G2/M cell cycle arrest. Regulates DNA repair through phosphorylation of BRCA2, enhancing the association of RAD51 with chromatin which promotes DNA repair by homologous recombination. Also stimulates the transcription of genes involved in DNA repair (including BRCA2) through the phosphorylation and activation of the transcription factor FOXM1. Regulates apoptosis through the phosphorylation of p53/TP53, MDM4 and PML. Phosphorylation of p53/TP53 at 'Ser-20' by CHEK2 may alleviate inhibition by MDM2, leading to accumulation of active p53/TP53. Phosphorylation of MDM4 may also reduce degradation of p53/TP53.	The presence of a CHEK2 mutation was protective against lung cáncer [odds ratio (OR) = 0.3; 95% confidence interval (CI) 0.2-0.5; P = 3 x 10(-8)]. Lung cáncers frequently possess other defects in genes in the DNA damage response pathway (e.g. p53 mutations) and have a high level of genotoxic DNA damage induced by tobacco smoke. We speculate that lung cáncer cells with impaired CHEK2 function undergo increased rates of cell death [261]. p.R474C mutation altered the tertiary structure of CHK2 by disrupting the salt bridge between p.R474 and p.E394 [262]. large-effect genome-wide associations for squamous lung cáncer with the rare variants CHEK2 p.Ile157Thr (rs17879961, OR = 0.38, P = 1.27 × 10−13) [263]. 70% of NSCLC, 33.34% of BC samples and 63.3% of control samples, the CHEK2- encoding gene contained mutations. Sequencing also confirmed these results. Statistical analysis showed that there was a direct relationship between the change in the position of 157 CHEK2 genes and metastatic NSCLC samples [264]. SNPs in CHEK2 are related to Chinese advanced NSCLC never-smoking female patients receiving platinum-based doublet chemotherapy in China [265]. The CHEK2 pathway may be important for the proliferation of lung adenocarcinoma, especially in tumors with chromosomal instability [266].
**COL5A1**	Type V collagen is a member of group I collagen (fibrillar forming collagen). It is a minor connective tissue component of nearly ubiquitous distribution. Type V collagen binds to DNA, heparan sulfate, thrombospondin, heparin, and insulin. Heparin-binding. Calcium, Metal-binding.	Knockdown of COL5A1 in human AD metastatic cells inhibited cell growth and invasion, and induced cell apoptosis. Notably, higher expression of COL5A1 was observed in the lung AD patients with recurrence and short survival [267]. Both in cell lines and in mouse model, the extracellular matrix receptors including the integrin (ITGA3 and ITGA2B), collagen (COL5A1), and laminin (LAMA5) were significantly inhibited by curcumin at messenger RNA and protein levels. Functional studies confirmed that curcumin not only induced A549 cell death but also repressed cell proliferation and migration by regulating extracellular matrix receptors IN NSCLC [268].
**CTHRC1**	May act as a negative regulator of collagen matrix deposition. Wnt-protein binding, frizzled binding	CTHRC1 induces non-small cell lung cáncer (NSCLC) invasion through upregulating MMP-7/MMP-9 [269]. Cthrc1 overexpression was significantly associated with differentiation (P = 0.039), tumor–node–metastasis (TNM) stage (P = 0.035), lymph node status (P = 0.001), and cigarette smoke (P = 0.037). Furthermore, it was shown that patients with high Cthrc1 expression had significantly poorer overall survival (OS) and disease-free survival (DFS; P = 0.004 and P = 0.010, respectively). Interestingly, high Cthrc1 expression was an independent prognostic factor for both OS and DFS (P = 0.010 and P = 0.005, respectively) only in NSCLCs with cigarette smoke[270]. CTHRC1 was evidently overexpressed in human NSCLC tissues and NSCLC cell lines at the protein and mRNA level. Ectopic up-regulation of CTHRC1 in cáncer cells resulted in elevated invasive and proliferative abilities, which were attenuated by the specific CTHRC1 siRNA. The biological effect of CTHRC1 on metastasis and proliferation was mediated by the activation of the Wnt/β-catenin pathway. Clinicopathologic analysis showed that CTHRC1 expression

		was significantly correlated with differentiation degree (p<0.001), clinical stage (p<0.001), T classification (p<0.001), lymph node metastasis (p=0.013) and distant metastasis (p<0.001). Kaplan-Meier analysis revealed that patients with high CTHRC1 expression had poorer overall survival rates than those with low CTHRC1 expression [271]. microRNA-30b inhibits cell invasion and migration through targeting collagen triple helix repeat containing 1 in non-small cell lung cáncer [272].
**CYP27A1**	Cytochrome P450 monooxygenase that catalyzes regio- and stereospecific hydroxylation of cholesterol and its derivatives. Hydroxylates (with R stereochemistry) the terminal methyl group of cholesterol side-chain in a three step reaction to yield at first a C26 alcohol, then a C26 aldehyde and finally a C26 acid. Regulates cholesterol homeostasis by catalyzing the conversion of excess cholesterol to bile acids via both the "neutral" (classic) and the "acid" (alternative) pathways. May also regulate cholesterol homeostasis via generation of active oxysterols, which act as ligands for NR1H2 and NR1H3 nuclear receptors, modulating the transcription of genes involved in lipid metabolism. Plays a role in cholestanol metabolism in the cerebellum. Similarly to cholesterol, hydroxylates cholestanol and may facilitate sterol diffusion through the blood-brain barrier to the systemic circulation for further degradation. Also hydroxylates retinal 7-ketocholesterol, a noxious oxysterol with pro-inflammatory and pro-apoptotic effects, and may play a role in its elimination from the retinal pigment epithelium. May play a redundant role in vitamin D biosynthesis.	CYP27B1 and CYP24A1 mRNA were upregulated in NSCLC compared with controls (P<0.05). However, no significant differences in CYP27A1 expression were observed between NSCLC and control. In addition, CYP24A1 expression was not associated with age, sex, smoking or TNM stage, but was associated with pathological type, differentiation and prognosis (P<0.05). CYP27B1 and CYP24A1 may be considered as independent prognostic factors of NSCLC and may be novel therapeutic targets to assist clinical diagnosis, treatment and prognosis of the disease [273]. Desmin-positive primary lung cáncers are rare. In the absence of skeletal muscle differentiation, desmin expression is observed exclusively in carcinomas with neuroendocrine differentiation, and most of them are high-grade carcinomas [274].
**DES**	Muscle-specific type III intermediate filament essential for proper muscular structure and function. Plays a crucial role in maintaining the structure of sarcomeres, inter-connecting the Z-disks and forming the myofibrils, linking them not only to the sarcolemmal cytoskeleton, but also to the nucleus and mitochondria, thus providing strength for the muscle fiber during activity.	Tumor cells were negative for CK20, thyroid transcriptional factor-1, CEA, S100 protein, desmin, α-smooth muscle actin, melanosome, CD34, p63, chromogranin, synaptophysin, CD56, KIT, and platelet-derived growth factor-α. It appeared that the present pulmonary PC is associated with adenocarcinoma or derived from sarcomatous transformation of adenocarcinoma [275]. INI-1, desmin, calretinin, and HMB-45 were negative as were markers for neuroendocrine differentiation [276].
**DOCK4**	Involved in regulation of adherens junction between cells. Plays a role in cell migration. Functions as a guanine núcleotide exchange factor (GEF), which activates Rap1 small GTPase by exchanging bound GDP for free GTP. Guanine-núcleotide releasing factor	TGF-β potently induces expression of DOCK4, but not other DOCK family members, via the Smad pathway and that DOCK4 induction mediates TGF-β's prometastatic effects by enhancing tumor cell extravasation. TGF-β-induced DOCK4 stimulates lung ADC cell protrusion, motility, and invasion without affecting epithelial-to-mesenchymal transition [277].
**DUOX1**	Generates hydrogen peroxide which is required for the activity of thyroid peroxidase/TPO and lactoperoxidase/LPO. Plays a role in thyroid hormones synthesis and lactoperoxidase-mediated antimicrobial defense at the surface of mucosa. May have its own peroxidase activity through its N-terminal peroxidase-like domain. Oxidoreductase, Peroxidase. Hydrogen peroxide, Thyroid hormone biosynthesis. Calcium, FAD, Flavoprotein, Metal-binding, NADP.	DUOX1 silencing in lung epithelial cáncer cells promotes features of EMT, and may be strongly associated with invasive and metastatic lung cáncer [278]. DUOX1 deficiency in lung cáncers promotes dysregulated EGFR signaling and enhanced GSTP1-mediated turnover of EGFR cysteine oxidation, which result in enhanced nuclear EGFR localization and tumorigenic properties [279]. Forskolin activates DUOX1 by means of phosphorylation mediated by protein kinase A [280]. Silencing of DUOX NADPH oxidases by promoter hypermethylation in lung cáncer [281].
**<u>E2F1</u>**	Transcription activator that binds DNA cooperatively with DP proteins through the E2 recognition site, 5'-TTTC[CG]CGC-3' found in the promoter region of a number of genes whose products are involved in cell cycle regulation or in DNA replication. The DRTF1/E2F complex functions in the control of cell-cycle progression from G1 to S phase. E2F1 binds preferentially RB1 in a cell-cycle dependent manner. It can mediate both cell proliferation and TP53/p53-dependent apoptosis. Blocks adipocyte differentiation by binding to specific promoters repressing CEBPA binding to its target gene promoters. Positively regulates transcription of RRP1B.	The expression levels of E2F1/2/3/5/6/7/8 were higher in lung adenocarcinoma and squamous cell lung carcinoma tissues than in lung tissues. Survival analysis using the Kaplan–Meier Plotter database revealed that the high transcription levels of E2F1/2/4/5/7/8 were associated with low relapse-free survival (RFS) in all of the patients with LC [282]. lncRNA-HIT interacts with E2F1 to regulate its target genes, such as Survivin, FOXM1, SKP2, NELL2 and DOK1, affecting the proliferation of NSCLC cells [283]. E2F1 could act as an oncogene being an essential actor of uncontrolled cellular proliferation, for example by counteracting the negative effects of cyclin-cdk inhibitors [284]. ANKRD22 promotes progression of non-small cell lung cáncer through transcriptional up-regulation of E2F1 [285].
**EBF1**	Transcriptional activator which recognizes variations of the palindromic sequence 5'-ATTCCCNNGGGAATT-3'. Metal-binding, Zinc.	EBF1 is an essential regulator of B-cell differentiation and maturation [286]. EBF1 inactivation could block these developmental processes, resulting in neoplastic phenotypes [287]. EBF1, in conjunction with E2A and Runx1, induces DNA demethylation and chromatin remodeling of the Cd79a and other B lineage target genes. This facilitates Pax5-mediated transactivation of these genes [288,289]. Overexpressing EBF1 suppressed PNO1 promoter activity and decreased PNO1 mRNA and protein, inhibiting cell proliferation and inducing cell apoptosis through the p53/p21 pathway [290]. EBF1-PDGFRB fusion results

		in loss of EBF1 function, multimerization and autophosphorylation of the fusion protein, activation of signal transducer and activator of transcription 5 (STAT5) signaling and gain of interleukin-7 (IL-7)-independent cell proliferation [291].
**FAM83A**	Probable proto-oncogene that functions in the epidermal growth factor receptor/EGFR signaling pathway. Activates both RAS/MAPK and PI3K/AKT/TOR signaling cascades downstream of EGFR. Required for the RAS/MAPK signaling cascade activation upon EGFR stimulation, it also activates both signaling cascades independently of EGFR activation.	A highly increased gene expression level of FAM83A (ø = 68-fold) and FAM83B (ø = 20-fold) which resulted in poor survival prognosis ($p < 0.0001$ and $p = 0.002$). Their expression was influenced by EGFR levels, pathway signaling, and mutation status. Both genes affected cell proliferation, and FAM83A depletion resulted in reduced migration and anchorage-independent growth [292]. overexpression of FAM83A-AS1 increased FAM38A protein levels and induced ERK1/2 phosphorylation downstream of FAM83A in cells. Finally, overexpression of FAM83A-AS1 promoted LUAD cell proliferation and invasion. In summary, lncRNA FAM83A-AS1 promotes LUAD by increasing FAM83A expression [293]. FAM83A was overexpressed in LUAD, and FAM83A overexpression could be used as an independent factor of poor prognosis in LUAD patients [294]. Among the ceRNAs identified, family with sequence similarity 83 member A (FAM83A), miR-34c-5p, KCNQ1OT1 and FLJ26245 were observed to be significantly associated with the overall survival of patients with LUAD. Of note, FAM83A has potential significance in drug resistance, and may present a candidate biomarker for the prognosis and treatment of patients with LUAD [295]. Four genes were selected for constructing the prognosis risk model, myosin IE (MYO1E), endoplasmic reticulum oxidoreductase 1α (ERO1L), C1q and tumor necrosis factor-related protein 6 (C1QTNF6) and family with sequence similarity 83, member A (FAM83A). The survival time of high- and low-risk groups in the validation set were significantly different. Functional enrichment revealed that the genes that interacted with MYO1E, ERO1L, C1QTNF6 and FAM83A separately were enriched in 'cell cycle regulation', 'synthesis and assembly of nucleic acids', 'histone modification and cell cycle progression' and 'cell secretion process'. The four-gene prognosis risk model could potentially be used for predicting the survival of patients with LAD [296].
**FANCG**	DNA repair protein that may operate in a postreplication repair or a cell cycle checkpoint function. May be implicated in interstrand DNA cross-link repair and in the maintenance of normal chromosome stability. Candidate tumor suppressor gene. DNA damage, DNA repair	This study demonstrates that inactivation of the FANC-BRCA pathway is relatively common in solid tumors and may be related to tobacco and alcohol exposure and survival of lung cancer patients [297].
**FAR2**	Catalyzes the reduction of saturated but not unsaturated C16 or C18 fatty acyl-CoA to fatty alcohols. A lower activity can be observed with shorter fatty acyl-CoA substrates. It may play a role in the production of ether lipids/plasmalogens and wax monoesters whose synthesis requires fatty alcohols as substrates. Oxidoreductase. Lipid biosynthesis, Lipid metabolism. NADP.	-
**FCN3**	May function in innate immunity through activation of the lectin complement pathway. Calcium-dependent and GlcNAc-binding lectin. Has affinity with GalNAc, GlcNAc, D-fucose, as mono/oligosaccharide and lipopolysaccharides from S.typhimurium and S.minnesota. Complement activation lectin pathway, Immunity, Innate immunity. Calcium, Lectin, Metal-binding.	The expression of genes FCN3, AGER and TMEM100 all significantly decreased in tumor tissues from patients with LUAD compared with normal tissues. Lowly expressed genes FCN3 and TMEM100 did not affect the survival of the patients in the samples tested [298]. Extracellular matrix (ECM)-associated components could be potential markers for better diagnosis and prognosis due to their differential expression in 1,943 primary NSCLC tumors as compared to 303 normal lung tissues. a 29-gene ECM-related prognostic and predictive indicator (EPPI). three genes, SPP1, MMP1, and S100A2, were strikingly upregulated and four genes, FCN3, HHIP, S100A12, and CPB2, were downregulated in both NSCLC and IPF. The patterns of the EPPI gene expression might be a critical gauge for impaired lung function, and 29 genes in the EPPI might form a network and collectively mediate tumor initiation. The EPPI signature may further be associated with the underlying mechanism of carcinogenesis such as epithelial-mesenchymal transition (EMT) or mesenchymal-epithelial transition (MET) [299]. FCN3 inhibits cell proliferation and induces apoptosis in lung cáncer cell lines [300].
**FERMT1**	Involved in cell adhesion. Contributes to integrin activation. When coexpressed with talin, it potentiates activation of ITGA2B. Required for normal keratinocyte proliferation. Required for normal polarization of basal keratinocytes in skin, and for normal cell shape. Required for normal adhesion of keratinocytes to fibronectin and laminin, and for normal keratinocyte migration to wound sites. May mediate TGF-beta 1 signaling in tumor progression.	Fermitin family member 1 (FERMT1, Kindlin-1) is an epithelial-specific regulator of integrin functions and is associated with Kindler syndrome, a genetic disorder characterized by skin blistering, atrophy, and photosensitivity. However, the possible role of kindlin-1 in cáncer remains unknown. Overexpression of kindlin-1 induced changes indicating epithelial-mesenchymal transition and transforming growth factor beta (TGFβ) signaling, constitutive activation of cell motility, and invasion [301]. Ectopic expression of Kindlin-1

		in non-small-cell lung cáncer cells inhibited in vitro cell migration and in vivo tumor growth, while Kindlin-2 promoted these functions. Mechanistically, Kindlin-1 prohibited epithelial to mesenchymal transition in non-small-cell lung cáncer cells, while Kindlin-2 enhanced epithelial to mesenchymal transition in these cells. Taken together, we demonstrated that Kindlin-1 and Kindlin-2 differentially regulate lung cáncer cell progression. Further, the expression levels of Kindlin-1 might be potentially used as a marker for lung cáncer differentiation and targeting Kindlin-2 might block the invasive growth of large cell lung cancer [302].
**FLRT3**	Functions in cell-cell adhesion, cell migration and axon guidance, exerting an attractive or repulsive role depending on its interaction partners. Plays a role in the spatial organization of brain neurons. Plays a role in vascular development in the retina. Plays a role in cell-cell adhesion via its interaction with ADGRL3 and probably also other latrophilins that are expressed at the surface of adjacent cells. Interaction with the intracellular domain of ROBO1 mediates axon attraction towards cells expressing NTN1. Mediates axon growth cone collapse and plays a repulsive role in neuron guidance via its interaction with UNC5B, and possibly also other UNC-5 family members. Promotes neurite outgrowth (in vitro). Mediates cell-cell contacts that promote an increase both in neurite number and in neurite length. Plays a role in the regulation of the density of glutamatergic synapses. Plays a role in fibroblast growth factor-mediated signaling cascades. Required for normal morphogenesis during embryonic development, but not for normal embryonic patterning. Required for normal ventral closure, headfold fusion and definitive endoderm migration during embryonic development. Required for the formation of a normal basement membrane and the maintenance of a normal anterior visceral endoderm during embryonic development.	Expression of some negative regulators of FGFR signaling [SPRY (Sprouty homologue) 2, SPRY4 and FLRT3 (fibronectin leucine-rich transmembrane protein 3)] was also increased in the PRMT5 shRNA expressing A549 cells [303]. List of genes with changed expression that are significantly under-represented in normal bronchus of smokers versus non-smokers (GO functional categories) [304].
**FOXF1**	Probable transcription activator for a number of lung-specific genes. Activator, DNA-binding. Transcription, Transcription regulation	Foxf1 deficiency altered expression of numerous genes including those regulating extracellular matrix remodeling (Timp3, Adamts9) and cell cycle progression (Cdkn1a, Cdkn2b, Cenpj, Tubb4a), which are critical for lung regeneration. Deletion of Foxf1 increased Timp3 mRNA and protein, decreasing MMP14 activity in regenerating lungs. ChIPseq analysis for FOXF1 and histone methylation marks identified DNA regulatory regions within the Cd44, Cdkn1a, and Cdkn2b genes, indicating they are direct FOXF1 targets. Thus FOXF1 stimulates lung regeneration following partial pneumonectomy via direct transcriptional regulation of genes critical for extracellular matrix remodeling and cell cycle progression [107]. MSCs fuse spontaneously with lung cáncer cells, and the latter is reprogrammed to slow growth and stem-like state. Transcriptome profiles reveal that lung cáncer cells are reprogrammed to a more benign state upon MSC fusion. We further identified FOXF1 as a reprogramming mediator that contributes not only to the reprogramming toward stemness but also to the p21-regulated growth suppression in fusion progeny [108]. The FENDRR is a long coding RNA (also named FOXF1-AS1) located in the vicinity of the protein-coding gene FOXF1 at 16q24.1 chromosomal region. The present study aimed to define the clinic pathological significance of the long-non-coding RNA FENDRR in lung adenocarcinomas. FENDRR expression measured by quantitative PCR was found significantly downregulated ($p<0.001$) in lung adenocarcinoma samples in comparison with their normal adjacent tissues (n=70) [109]. Alveolar Capillary Dysplasia with Misalignment of Pulmonary Veins (ACDMPV) is a developmental disorder of the lungs, primarily affecting their vasculature. FOXF1 haploinsufficiency due to heterozygous genomic deletions and point mutations have been reported in most patients with ACDMPV. The majority of mice with heterozygous loss-of-function of Foxf1 exhibit neonatal lethality with evidence of pulmonary hemorrhage in some of them. By comparing transcriptomes of human ACDMPV lungs with control lungs using expression arrays, we found that several genes and pathways involved in lung development, angiogenesis, and in pulmonary hypertension development, were deregulated. Similar transcriptional changes were found in lungs of the postnatal day 0.5 Foxf1+/- mice when compared to their wildtype littermate controls; 14 genes, COL15A1, COL18A1, COL6A2, ESM1, FSCN1, GRINA, IGFBP3, IL1B, MALL, NOS3, RASL11B, MATN2, PRKCDBP, and SIRPA, were found common to both ACDMPV and Foxf1

		heterozygous lungs [305]. Loss of long noncoding RNA FOXF1-AS1 regulates epithelial-mesenchymal transition, stemness and metastasis of non-small cell lung cáncer cells [110]. Long non-coding RNA FOXF1 adjacent non-coding developmental regulatory RNA inhibits growth and chemotherapy resistance in non-small cell lung cáncer [111].
**FOXF2**	Probable transcription activator for a number of lung-specific genes. Mediates up-regulation of the E3 ligase IRF2BPL and drives ubiquitination and degradation of CTNNB1. . Activator, DNA-binding. Transcription, Transcription regulation	FOXF2 expression is a predictive factor for poor prognosis of patients with NSCLC, especially in stage I NSCLC [306]. The miR-200 family and the miR-183~96~182 cluster target Foxf2 to inhibit invasion and metastasis in lung cáncers [307].
**FRMD4A**	Scaffolding protein that regulates epithelial cell polarity by connecting ARF6 activation with the PAR3 complex. Plays a redundant role with FRMD4B in epithelial polarization. May regulate MAPT secretion by activating ARF6-signaling.	Mice were sacrificed after 10 days and their lungs scanned. As in the case of skin and tongue tumors, FRMD4A knockdown reduced tumor growth [308]. This fusion retains the RET kinase domain, which spans amino acid 724 to amino acid 1016, occurs at a recurrent RET fusion breakpoint (intron 11), and retains the N-terminal coiled-coil domain of FRMD4A, allowing constitutive dimerization of FRMD4A-RET and subsequent activation of the RET kinase [309].
**GATA6**	Transcriptional activator Regulates SEMA3C and PLXNA2. Involved in gene regulation specifically in the gastric epithelium. May regulate genes that protect epithelial cells from bacterial infection. Involved in bone morphogenetic protein (BMP)-mediated cardiac-specific gene expression. Binds to BMP response element (BMPRE) DNA sequences within cardiac activating regions. Activator, DNA-binding. Transcription, Transcription regulation. Metal-binding, Zinc. GATA6 suppression enhances lung specification from human pluripotent stem cells [310].	The Em/Ad expression ratios of GATA6 and NKX2-1 detected exhaled breath condensates (EBCs) were combined using linear kernel support vector machines (SVM) into the LC score, which can be used for LC detection. LC score-based diagnosis achieved a high performance in an independent validation cohort [311]. miR-196b promotes lung cáncer cell migration and invasion through the targeting of GATA6 [312]. GATA6 could enhance autophagy activity contributing to tyrosine kinase inhibitor (TKI) resistance. Targeting GATA6 and autophagy together with TKI may be promising to overcome drug resistance in NSCLC [313]. Retinoic Acid affects Lung Adenocarcinoma growth by inducing differentiation via GATA6 activation and EGFR and Wnt inhibition. GATA6 activation is necessary for EGFR and Wnt inhibition, thus leading to 1) increased differentiation and 2) loss of proliferation [112]. GATA6 and HOPX are critical nodes in a lineage-selective pathway that directly links effectors of airway epithelial specification to the inhibition of metastasis in the lung ADC subtype [113].
**GRASP**	Plays a role in intracellular trafficking and contributes to the macromolecular organization of group 1 metabotropic glutamate receptors (mGluRs) at synapses.	-
**GREM1**	Cytokine may play an important role during carcinogenesis and metanephric kidney organogenesis, as a BMP antagonist required for early limb outgrowth and patterning in maintaining the FGF4-SHH feedback loop. Down-regulates the BMP4 signaling in a dose-dependent manner. Antagonist of BMP2 inhibits BMP2-mediated differentiation of osteoblasts (in vitro). Acts as an inhibitor of monocyte chemotaxis. Can inhibit the growth or viability of normal cells but not transformed cells when overexpressed.	Lung adenocarcinoma but not squamous cell carcinoma shows a significant increase in Gremlin expression by mRNA and protein level. Lung fibroblast and epithelial cell lines transfected with GREM1 show significantly increased cell proliferation [314]. gremlin was increased in the walls of small intrapulmonary vessels in idiopathic pulmonary arterial hypertension and the rare heritable form of pulmonary arterial hypertension in a distribution suggesting endothelial localization [315]. High expression of Gremlin-1 by fibroblasts stimulates proliferation of lung adenocarcinoma tumor cells [316]. Gene clusters generated from hierarchical clustering using validation cohorts probed with the full-gene platforms (Discovery set, GSE3141, GSE37745, and GSE30219) revealed that high-risk groups associated with worse survival displayed consistently elevated expressions of collagens (COL10A1, COL11A1), matrix metallopeptidases (MMP1, MMP12), secreted factors (S100A2), glycoproteins (CTHRC1, SPP1), and ECM-affiliated proteins, or genes encoding proteins affiliated structurally or functionally to ECM proteins (GREM1) and low expressions of surfactant proteins (SFTPC, SFTPA2, SFTPD), secreted proteins (CHRDL1, WIFI), ECM-regulated genes (CPB2, MAMDC2, HHIP, LPL, CD36, ADAMTS8), collagen (COL6A6), ECM-affiliated proteins (FCN3), ECM glycoproteins (TNNC1, ABI3BP), and proteoglycan (OGN) [299].
**HOXA5**	Sequence-specific transcription factor which is part of a developmental regulatory system that provides cells with specific positional identities on the anterior-posterior axis. Also binds to its own promoter. Binds specifically to the motif 5'-CYYNATTA[TG]Y-3'. Developmental protein, DNA-binding. Transcription, Transcription regulation. ablation of Hoxa5 in mesenchyme perturbed trachea development, lung epithelial cell differentiation and lung growth [317].	HOXA5 indicates poor prognosis and suppresses cell proliferation by regulating p21 expression in non-small cell lung cáncer [318]. HOXA5 and p53 cooperate to suppress lung cáncer cell invasion and serve as good prognostic factors in non-small cell lung cáncer [319]. HOXA5 Inhibits Metastasis via Regulating Cytoskeletal remodeling and Associates with Prolonged Survival in Non-Small-Cell Lung Carcinoma [320]. Long non-coding RNA 00312 regulated by HOXA5 inhibits tumor proliferation and promotes apoptosis in Non-small cell lung cáncer [321].

**IGSF10**	Involved in the control of early migration of neurons expressing gonadotropin-releasing hormone (GNRH neurons). May be involved in the maintenance of osteochondroprogenitor cells pool.	Down-regulated in adenocarcinoma [219,322].
**KCNK3**	pH-dependent, voltage-insensitive, background potassium channel protein. Rectification direction results from potassium ion concentration on either side of the membrane. Acts as an outward rectifier when external potassium concentration is low. When external potassium concentration is high, current is inward. Ion channel, Potassium channel. Ion transport, Potassium transport, Transport. Potassium.	The two-pore domain K+ (K2P) channel TASK-1 (KCNK3) knockdown by siRNA significantly enhanced apoptosis and reduced proliferation in A549 cells [323].
**KCNT2**	Outward rectifying potassium channel. Produces rapidly activating outward rectifier K+ currents. Activated by high intracellular sodium and chloride levels. Channel activity is inhibited by ATP and by inhalation anesthetics, such as isoflurane. Inhibited upon stimulation of G-protein coupled receptors, such as CHRM1 and GRM1. Ion channel, Potassium channel. Ion transport, Potassium transport, Transport. ATP-binding, Núcleotide-binding, Potassium.	Potential mechanism of resistance to afatinib treatment might be related to mutations in HLA-DRB1, AQP7, TP53, HLA-DRB5, PRSS3, USH2A, KCNT2 and CNN2 and to mutations in genes of the Wnt and PI3K-AKT pathways [324].
**LOXL2**	Mediates the post-translational oxidative deamination of lysine residues on target proteins leading to the formation of deaminated lysine (allysine). Acts as a transcription corepressor and specifically mediates deamination of trimethylated 'Lys-4' of histone H3 (H3K4me3), a specific tag for epigenetic transcriptional activation. Shows no activity against histone H3 when it is trimethylated on 'Lys-9' (H3K9me3) or 'Lys-27' (H3K27me3) or when 'Lys-4' is monomethylated (H3K4me1) or dimethylated (H3K4me2). Also mediates deamination of methylated TAF10, a member of the transcription factor IID (TFIID) complex, which induces release of TAF10 from promoters, leading to inhibition of TFIID-dependent transcription . LOXL2-mediated deamination of TAF10 results in transcriptional repression of genes required for embryonic stem cell pluripotency including POU5F1/OCT4, NANOG, KLF4 and SOX2. Involved in epithelial to mesenchymal transition (EMT) via interaction with SNAI1 and participates in repression of E-cadherin CDH1, probably by mediating deamination of histone H3. During EMT, involved with SNAI1 in negatively regulating pericentromeric heterochromatin transcription.	Lysyl Oxidase-like Protein LOXL2 Promotes Lung Metastasis of Breast Cáncer [325] downregulation of miR-29a in clinical specimens of IPF and lung cáncer. Restoration of miR-29a suppressed cáncer cell aggressiveness and fibroblast migration. A combination of gene expression data and in silico analysis showed that a total of 24 genes were putative targets of miR-29a. Among them, lysyl oxidase-like 2 (LOXL2) and serpin peptidase inhibitor clade H, member 1 (SERPINH1) were direct targets of miR-29a by luciferase reporter assays. The functions of LOXL2 and SERPINH1 contribute significantly to collagen biosynthesis. Overexpression of LOXL2 and SERPINH1 was observed in clinical specimens of lung cáncer and fibrotic lesions. Downregulation of miR-29a caused overexpression of LOXL2 and SERPINH1 in lung cáncer and IPF, suggesting that these genes are involved in the pathogenesis of these two diseases [326]. Compared to controls, LOXL2 levels were significantly ($p < 0.001$–0.05) elevated in serum from patients with breast, colorectal, lung, ovarian and pancreatic cáncer (mean range: 49–84 ng/mL), but not in prostate cáncer (mean: 36 ng/mL) and malignant melanoma patients (41 ng/mL). Serum LOXL2 was elevated in IPF patients compared to healthy controls (mean: 76.5 vs 46.8 ng/mL; $p > 0.001$)[871] [327]. E47 or LOXL2 silencing reduces tumor growth and decreases lung metastasis [328].
**LRRC32**	Key regulator of transforming growth factor beta (TGFB1, TGFB2 and TGFB3) that controls TGF-beta activation by maintaining it in a latent state during storage in extracellular space. Associates specifically via disulfide bonds with the Latency-associated peptide (LAP), which is the regulatory chain of TGF-beta, and regulates integrin-dependent activation of TGF-beta. Able to outcompete LTBP1 for binding to LAP regulatory chain of TGF-beta. Controls activation of TGF-beta-1 (TGFB1) on the surface of activated regulatory T-cells (Tregs). Required for epithelial fusion during palate development by regulating activation of TGF-beta-3 (TGFB3). Growth factor binding	Compared with the control group, the expression of GARP in Tregs was increased in tumor tissues of lung cáncer patients and was associated with lymph node metastasis, distant metastasis and clinical stage [329].
**MAMDC2**	MAMDC2 (MAM Domain Containing 2) is a Protein Coding gene. Diseases associated with MAMDC2 include Kabuki Syndrome 1 and West Syndrome. Gene Ontology (GO) annotations related to this gene include glycosaminoglycan binding. An important paralog of this gene is MAMDC4.	Down-regulated (squamous cell carcinoma) [219]. Top hypermethylated and down-regulated genes in lung adenocarcinoma [242].
**MEF2A**	Transcriptional activator which binds specifically to the MEF2 element, 5'-YTA[AT]4TAR-3', found in numerous muscle-specific genes. Also involved in the activation of numerous growth factor- and stress-induced genes. Mediates cellular functions not only in skeletal and cardiac muscle development, but also in neuronal differentiation and survival. Plays diverse roles in the control of cell growth, survival and apoptosis via p38 MAPK signaling in muscle-specific and/or growth factor-related transcription. In cerebellar granule neurons, phosphorylated and sumoylated MEF2A represses transcription of NUR77 promoting synaptic differentiation. Associates with chromatin to the ZNF16 promoter. Activator, Developmental protein, DNA-binding. Apoptosis, Differentiation, Neurogenesis, Transcription, Transcription regulation.	Inflammatory conditions led to increased MEF2D expression, which might further contribute to the development of lung cáncer through influencing cáncer microenvironment and cell bio-behaviors, including proliferation, differentiation, and movement. MEF2D might be a potential biomarker during chronic inflammation-lung cáncer transition, predicting the risk of lung cáncer among patients with chronic respiratory diseases [330]. oleanolic acid is a new type of anti-tumor drug that suppresses proliferation of lung cáncer cells via inhibition of MEF2D expression [331]. miR-218 suppressed the growth of lung carcinoma by reducing MEF2D expression [332].
**MND1**	Required for proper homologous chromosome pairing and efficient crossover and intragenic recombination during meiosis. Stimulates both DMC1- and RAD51-mediated homologous strand assimilation, which is required for the resolution of meiotic double-strand breaks. DNA-binding. DNA recombination, Meiosis	MND1 was negatively associated with the overall survival of patients with LUSC. MND1 was upregulated in LUAD and LUSC. MND1 and SCGB1A1 were significantly different among different stages of LUSC [691]. MND1 gene binds to PSMC3 interacting protein to form stable heterodimer complexes that bind to DNA and stimulate the activities of RAD51 recombinase and DNA meiotic recombinase 1, which are required for meiotic recombination [333].

**NFE2L2**	Transcription activator that binds to antioxidant response (ARE) elements in the promoter regions of target genes. Important for the coordinated up-regulation of genes in response to oxidative stress and the regulation of cellular redox conditions. May be involved in the transcriptional activation of genes of the beta-globin cluster by mediating enhancer activity of hypersensitive site 2 of the beta-globin locus control region. Activator, DNA-binding. Host-virus interaction, Transcription, Transcription regulation.	NFE2L2 encodes Nrf2, a transcription factor involved in the oxidative stress response which is targeted for degradation by Keap1. The KEAP1/NRF2 pathway has been widely investigated in tumors since it was implicated in cáncer cells survival and therapies resistance. In lung tumors the deregulation of this pathway is mainly related to point mutations of KEAP1 and NFE2L2 genes and KEAP1 promoter hypermethylation, but these two genes have been rarely investigated in low/intermediate grade neuroendocrine tumors of the lung [334]. a prevalence of KEAP1-NFE2L2 (31%) alterations in tumors with high neuroendocrine gene expression, mainly co-occurring with STK11 and KRAS genes to exert a synergic role of tumorigenesis enhancement and cáncer progression [335]. Targeting NFE2L2 mutations in advanced squamous cell lung cáncers with the TORC1/2 inhibitor TAK228 [336]. A risk scoring system based on NFE2L2 gene expression profiling and designated 50 tumor-associated genes as the NFE2L2-associated molecular signature (NAMS). NAMS predicts clinical outcome in the training cohort and in 12 out of 20 validation cohorts. Cox proportional hazard regressions indicate that NAMS is a robust prognostic gene signature, independent of other clinical and pathological factors including patient age, gender, smoking, gene alteration, MYC level, and cáncer stage [337]. Nrf2 could prevent cells from undergoing oncogenesis as a tumor suppressor, while it could also promote cáncer progression and resistance to chemotherapeutic drugs as an oncogene, depending on the different stages of tumor progression. Target Nrf2 signaling by specific chemicals showed it could prevent tumor growth or combat chemoresistance [338]. A Phase II Study of MLN0128 to Treat Advanced Squamous Cell Lung Cáncer. a novel immune signature, characterized by the infiltration of tumor-promoting immune cells, elevated cytokines, and increased expression of immune response genes in the lungs and tumors of Nrf2 KO mice [339]. For NSCLC patients with mutations in *EGFR*, co-mutations in *KEAP1/NFE2L2/CUL3* are associated with significantly decreased time to treatment failure. Our results suggest that these mutations represent a mechanism of intrinsic resistance to TKI treatment [340].
**NFIL3**	Acts as a transcriptional regulator that recognizes and binds to the sequence 5'-[GA]TTA[CT]GTAA[CT]-3', a sequence present in many cellular and viral promoters. Represses transcription from promoters with activating transcription factor (ATF) sites. Represses promoter activity in osteoblasts. Represses transcriptional activity of PER1. Represses transcriptional activity of PER2 via the B-site on the promoter. Activates transcription from the interleukin-3 promoter in T-cells. Competes for the same consensus-binding site with PAR DNA-binding factors (DBP, HLF and TEF). Component of the circadian clock that acts as a negative regulator for the circadian expression of PER2 oscillation in the cell-autonomous core clock. Protects pro-B cells from programmed cell death. Represses the transcription of CYP2A5. Positively regulates the expression and activity of CES2 by antagonizing the repressive action of NR1D1 on CES2. Activator, DNA-binding, Repressor. Biological rhythms, Transcription, Transcription regulation.	Nfil3 was almost ubiquitously expressed and was present at relatively high levels in lung, liver, and BM [341].
**NOSTRIN**	Multivalent adapter protein which may decrease NOS3 activity by inducing its translocation away from the plasma membrane. Endocytosis	NOSTRIN mRNA is abundant in highly vascularized tissues such as placenta, kidney, lung, and heart, and NOSTRIN protein is expressed in vascular endothelial cells [342]. NOSTRIN expression leading to deactivation of the NFκB pathway, in turn triggering an anti-angiogenic cascade, might inhibit tumorigenesis and cáncer progression [343].
**NPM3**	Plays a role in the regulation of diverse cellular processes such as ribosome biogenesis, chromatin remodeling or protein chaperoning. Modulates the histone chaperone function and the RNA-binding activity of núcleolar phosphoprotein B23/NPM. Efficiently mediates chromatin remodeling when included in a pentamer containing NPM3 and NPM. Chaperone	Strong c-Myc DNA binding activity at promoter sites of Npm1 and Npm3 was observed in EMSA, as well as ChIP assays and the results are confirmed in independent ChIP-seq experiments; equally our gene reporter assays confirm Npm3 to be activated by c-Myc.Gene expression signature in c-Myc-induced lung papillary adenocarcinoma: differentially expressed genes involved in stimulation of cell proliferation and growth [344].
**NR4A2**	Transcriptional regulator which is important for the differentiation and maintenance of meso-diencephalic dopaminergic (mdDA) neurons during development. It is crucial for expression of a set of genes such as SLC6A3, SLC18A2, TH and DRD2 which are essential for development of mdDA neurons.	Transcriptionally regulate cell proliferation, apoptosis, inflammation, neuronal development, and carcinogenesis [345,346]. Overexpression of NR4A2 blocked the induction of p53 target genes, including mir-34a, which could rescue cells from p53-induced inhibition of proliferation [347].
**NUF2**	Acts as a component of the essential kinetochore-associated NDC80 complex, which is required for chromosome segregation and spindle checkpoint activity. Required for kinetochore integrity and the	Aberrant centromere and kinetochore function causes CIN through chromosome missegregation, leading to aneuploidy, rearrangements and micronucleus formation. Here

	organization of stable microtubule binding sites in the outer plate of the kinetochore. The NDC80 complex synergistically enhances the affinity of the SKA1 complex for microtubules and may allow the NDC80 complex to track depolymerizing microtubules. Cell cycle, Cell division, Mitosis.	we develop a Centromere and kinetochore gene Expression Score (CES) signature that quantifies the centromere and kinetochore gene misexpression in cáncers. High CES values correlate with increased levels of genomic instability and several specific adverse tumor properties, and prognosticate poor patient survival for breast and lung cáncers, especially early-stage tumors. They also signify high levels of genomic instability that sensitize cáncer cells to additional genotoxicity. misregulation of CEN/KT genes causes chromosomal abnormalities that contribute to tumorigenesis, and can be used as a biomarker for predicting patient prognosis and response to therapy. CENP-W, -L, -K, SPC24 and NUF2 were included in the final core CEN/KT gene list [348]. Five human genes as candidate regulators of CNA in human lung adenocarcinoma: MMP13, NUF2, CCL22, ZNF366, and GPR114. High expression of MMP13, CCL22, ZNF366, and GPR114 was correlated with low Copy Number Alterations (CNA), suggesting their role in suppressing CIN. High expression of NUF2 was correlated with high CNA, suggesting its role in increasing Chromosome instability (CIN). NUF2 (yeast NUF2 Kinetochore Protein homolog) is involved in the mitotic process, the misregulation of which directly leads to CIN [349].
**NXF3**	May function as a tissue-specific nuclear mRNA export factor. RNA-binding. mRNA transport, Transport.	Strongest positive and negative correlation between the most expressed messenger RNA (top upregulated and downregulated genes) and miR-155/miR-21/miR-4459 lung expression [350].
**PAK6**	Serine/threonine protein kinase that plays a role in the regulation of gene transcription. The kinase activity is induced by various effectors including AR or MAP2K6/MAPKK6. Phosphorylates the DNA-binding domain of androgen receptor/AR and thereby inhibits AR-mediated transcription. Inhibits also ESR1-mediated transcription. May play a role in cytoskeleton regulation by interacting with IQGAP1. May protect cells from apoptosis through phosphorylation of BAD. Kinase, Serine/threonine-protein kinase, Transferase. ATP-binding, Núcleotide-binding	hyperphosphorylation of S560 within the conserved kinase domain of PAK6. Activation of PAK6 is associated with various processes in cáncer including metastasis. Mechanistic studies revealed that inhibition of PAK6 led to reduction in cell proliferation, migration and invasion of the cigarette smoke treated cells. Further, siRNA mediated silencing of PAK6 resulted in decreased invasive abilities in a panel of non-small cell lung cáncer (NSCLC) cells [351]. hsa-mir-183-3p affected the expression of other genes in this pathway, including PAK1, PAK4, PAK6, PIK3CD, PIK3R3, AKT2, ERBB2, PRKCA (1249), and CAMK2G (4297). The genes in the ERBB signaling pathway have three different functional types: one group causes death (GSK3B, PRKCA, ERBB2, SOS2, CAMK2G, PAK1, AKT2, MYC, PAK4, PAK6, and ABL2); a second promotes survival (PLCG1, PIK3CD, and PIK3R3); and a third group has no relationship to survival (CRK, MAPK10, CRK, and EREG) [352].
**PBX1**	Binds the sequence 5'-ATCAATCAA-3'. Acts as a transcriptional activator of PF4 in complex with MEIS1. Converted into a potent transcriptional activator by the (1;19) translocation. May have a role in steroidogenesis and, subsequently, sexual development and differentiation. Isoform PBX1b as part of a PDX1:PBX1b:MEIS2b complex in pancreatic acinar cells is involved in the transcriptional activation of the ELA1 enhancer; the complex binds to the enhancer B element and cooperates with the transcription factor 1 complex (PTF1) bound to the enhancer A element. Probably in complexity with MEIS2, it is involved in transcriptional regulation by KLF4. Acts as a transcriptional activator of NKX2-5 and a transcriptional repressor of CDKN2B. Together with NKX2-5, it is required for spleen development through a mechanism that involves CDKN2B repression.	Pbx1 overexpression repressed the proliferation of lung cáncer cells and inhibited DNA synthesis [353]. E2A-PBX1 fusion gene caused by t(1;19)(q23;p13) may be a common genetic change in AIS and a survival determinant for female AIS patients at an early stage [354]. Downregulated in carcinoma-associated fibroblasts (CAF) [355].
**PDE5A**	Plays a role in signal transduction by regulating the intracellular concentration of cyclic núcleotides. This phosphodiesterase catalyzes the specific hydrolysis of cGMP to 5'-GMP. Specifically regulates nitric-oxide-generated cGMP. Allosteric enzyme, Hydrolase. cGMP, cGMP-binding, Metal-binding, Núcleotide-binding.	PDE5A is the modulation of vascular tone via regulation of intracellular cGMP and calcium levels, particularly in the lung and penis [356]. PDE5 as an important target for CSC maintenance in different tumor cell types [357]. Wharton et al demonstrated a significant upregulation of PDE5A in lungs from patients with PAH [358].
**PRKCE**	Calcium-independent, phospholipid- and diacylglycerol (DAG)-dependent serine/threonine-protein kinase that plays essential roles in the regulation of multiple cellular processes linked to cytoskeletal proteins, such as cell adhesion, motility, migration and cell cycle, functions in neuron growth and ion channel regulation, and is involved in immune response, cáncer cell invasion and regulation of apoptosis. Mediates cell adhesion to the extracellular matrix via integrin-dependent signaling, by mediating angiotensin-2-induced activation of integrin beta-1 (ITGB1) in cardiac fibroblasts. Phosphorylates MARCKS, which phosphorylates and activates PTK2/FAK, leading to the spread of cardiomyocytes. Involved in the control of the directional transport of ITGB1 in mesenchymal cells by phosphorylating vimentin (VIM), an intermediate filament (IF) protein. In epithelial cells, it associates with and phosphorylates keratin-8 (KRT8), which induces targeting of desmoplakin at desmosomes and regulates cell-cell contact. Phosphorylates IQGAP1, which binds to	Protein kinase C-epsilon promotes survival of lung cáncer cells by suppressing apoptosis through dysregulation of the mitochondrial caspase pathway [359]. Non-Small Cell Lung Carcinoma Cell Motility, Rac Activation and Metastatic Dissemination Are Mediated by Protein Kinase C Epsilon [360]. Genetic ablation of PKCε impairs chemically-induced lung carcinogenesis. PKCε is a novel effector of KRAS in lung cáncer that may represent a promising target for disease treatment [361]. a variant SCLC cell line expresses a constitutively active catalytic fragment of PKC epsilon. Regulation by 12-O- tetradecanoyl-13-acetate or GRP via de novo protein synthesis suggests a novel mechanism of control of PKC diversity with implications for small cell lung cáncer and possibly other malignancies [362]. Inhibition of the PKCε pathway using a kinase-inactive, dominant-negative PKCε,

	CDC42, mediating epithelial cell-cell detachment prior to migration. In HeLa cells, it contributes to hepatocyte growth factor (HGF)-induced cell migration, and in human corneal epithelial cells, plays a critical role in wound healing after activation by HGF. During cytokinesis, forms a complex with YWHAB, which is crucial for daughter cell separation, and facilitates abscission by a mechanism which may implicate the regulation of RHOA. In cardiac myocytes, it regulates myofilament function and excitation coupling at the Z-lines, where it is indirectly associated with F-actin via interaction with COPB1.	PKCε(KR), led to a significant inhibition of proliferation and anchorage-independent growth of human NSCLC cells in a p53-independent manner. This was accompanied by a specific induction of the cyclin-dependent kinase (cdk) inhibitor p21/Cip1 but not p27/Kip1 [363]. Targeting PKCε by miR-143 regulates cell apoptosis in lung cáncer [364]. Persistent cell motility requires the formation of stable lamellae at the leading edge of a migrating cell. Here, we report that the tight junction protein zonula occludens-1 (ZO-1) preferentially interacts with α5β1 integrin at the lamellae of migrating cells. Disruption of ZO-1 binding to an internal PDZ-binding motif in the α5 cytoplasmic tail prevented the polarized localization of ZO-1 and α5 at the leading edge. Furthermore, silencing of α5 integrin inhibited migration and invasion of lung cáncer cells, and silencing of ZO-1 resulted in increased Rac activity and reduced directional cell motility. The formation of the α5–ZO-1 complex was dependent on PKCε: Phosphorylation of ZO-1 at serine-168 regulated the subcellular localization of ZO-1 and thus controlled its association with α5 integrin [365]. TACE/ADAM-17 Phosphorylation by PKC-Epsilon Mediates Premalignant Changes in Tobacco Smoke-Exposed Lung Cells [366].
**RHOJ**	Plasma membrane-associated small GTPase specifically involved in angiogenesis . Required for endothelial cell migration during vascular development via its interaction with GLUL. Elicits the formation of F-actin-rich structures, thereby regulating endothelial cell migration. Angiogenesis. GTP-binding, Núcleotide-binding	In the LLC (Lewis lung carcinoma) tumors, RhoJ deletion inhibits tumor growth, neovessel formation, and metastasis [367]. Those Gene Body loci with concordant methylation and expression included: hypermethylated/increased GE: FERMT1, SLC7A5, FAP, KRT15, ETV4, TFAP2a TPX2, FOXM1; hypomethylated/decreased GE: AGBL1, RHOJ, LDB2, GHR, ITGA8, ABCB1, SEMA5A, GPM6A [368]. RHOJ. The expression of these three genes were all downregulated in LUAD [369].
**RPGR**	Could be a guanine-núcleotide releasing factor. Plays a role in ciliogenesis. Probably regulates cilia formation by regulating actin stress filaments and cell contractility. Plays an important role in photoreceptor integrity. May play a critical role in spermatogenesis and in intraflagellar transport processes.	-
**RSPO1**	Activator of the canonical Wnt signaling pathway by acting as a ligand for LGR4-6 receptors. Upon binding to LGR4-6 (LGR4, LGR5 or LGR6), LGR4-6 associate with phosphorylated LRP6 and frizzled receptors that are activated by extracellular Wnt receptors, triggering the canonical Wnt signaling pathway to increase expression of target genes. Also regulates the canonical Wnt/beta-catenin-dependent pathway and non-canonical Wnt signaling by acting as an inhibitor of ZNRF3, an important regulator of the Wnt signaling pathway. Acts as a ligand for frizzled FZD8 and LRP6. May negatively regulate the TGF-beta pathway.	Rspo2 homozygous mutant mice die immediately after birth and display multiple defects including limb defects, craniofacial and laryngeal-tracheal malformation, lung hypoplasia, and kidney malformationsIn agreement with AER and BA1, Rspo2 acts through the LRP6-mediated WNT/β-catenin signaling during lung development as Rspo2 mutant lungs showed reduced WNT/β-catenin signaling and Rspo2 and Lrp6 compound mutant mice exhibited synergistic hypoplasia phenotype [370]. RSPO1, RSPO2 and RSPO3 were significantly lower in patients with lung cáncer. In the survival analysis, increased mRNA expression levels of RSPO1, RSPO2 and RSPO3 were associated with increased survival in patients with lung adenocarcinomas [355].
**<u>RUNX1</u>**	Essential for the development of normal hematopoiesis. Acts synergistically with ELF4 to transactivate the IL-3 promoter and with ELF2 to transactivate the BLK promoter. Inhibits KAT6B-dependent transcriptional activation. Involved in lineage commitment of immature T cell precursors. CBF complexes repress ZBTB7B transcription factor during cytotoxic (CD8+) T cell development. They bind to RUNX-binding sequence within the ZBTB7B locus acting as transcriptional silencer and allowing for cytotoxic T cell differentiation. CBF complexes binding to the transcriptional silencer is essential for recruitment of nuclear protein complexes that catalyze epigenetic modifications to establish epigenetic ZBTB7B silencing. Controls the energy and suppressive function of regulatory T-cells by associating with FOXP3. Activates the expression of IL2 and IFNG and down-regulates the expression of TNFRSF18, IL2RA and CTLA4, in conventional T-cells. Positively regulates the expression of RORC in T-helper 17 cells.	Loss of RUNX1 resulted in enhanced proliferation, migration, and invasion. RUNX1 depletion also resulted in increased mRNA expression of E2F1 and multiple E2F1 target genes, implicating the loss of RUNX1 as driver of lung adenocarcinoma aggression, potentially through deregulation of the E2F1 pathway [371]. Tet-inducible shRNA mediated RUNX1 knockdown in wild-type EGFR cell line LU99A led to the increased expression of Mig6 and decreased expression of phosphorylated form of EGFR as well as deactivation of EGFR/ERK pathway. RUNX1 knockdown subsequently induced apoptosis in LU99A cells. RUNX1 overexpression promoted cell proliferation and decreased expression of Mig6 and increased phosphorylated form of EGFR. Notably Mig6 knockdown in RUNX1 knockdown LU99A cells increased their proliferation rates, indicating that Mig6 plays an important role in RUNX1-mediated pro-oncogenic pathway [372].
**SGCA**	Component of the sarcoglycan complex, a subcomplex of the dystrophin-glycoprotein complex which forms a link between the F-actin cytoskeleton and the extracellular matrix.	.
**SLC6A4**	Serotonin transporter whose primary function in the central nervous system involves the regulation of serotonergic signaling via transport of serotonin molecules from the synaptic cleft back into the presynaptic terminal for re-utilization.	Down-regulated (adenocarcinoma) [219]. SLC6A4 could be modified to prevent COPD and treat the depressive symptoms of COPD [373].
**SLCO2A1**	May mediate the release of newly synthesized prostaglandins from cells, the transepithelial transport of prostaglandins, and the clearance of prostaglandins from the circulation. Transports PGD2, as well as PGE1, PGE2 and PGF2A .	Prostaglandin transporter, SLCO2A1, mediates the invasion and apoptosis of lung cáncer cells via the PI3K/AKT/mTOR pathway [374].

**STXBP6**	Forms non-fusogenic complexes with SNAP25 and STX1A and may thereby modulate the formation of functional SNARE complexes and exocytosis.	overexpressed STXBP6 in A549 and H1299 cells significantly decreased cell proliferation, colony formation, and migration, and increased apoptosis. Finally, significantly lower survival rates ($P < 0.05$) were observed when expression levels of STXBP6 were low. Our results provide a basis for the genetic etiology of lung adenocarcinoma by demonstrating the possible role of hypermethylation of STXBP6 in poor clinical outcomes in lung cáncer patients [375]. The methylation and expression validation results identified 4 candidate genes (STXBP6, BCL6B, FZD10, and HSPB6) that were significantly hypermethylated and downregulated in most of the tumor tissues compared with the noncáncerous lung tissues [376].
**TM6SF1**	May function as sterol isomerase.	Top hypermethylated and down-regulated genes in lung adenocarcinoma [242]. List of DEGs in lung SCC [248].
**TMPRSS4**	Probable protease. Seems to be capable of activating ENaC. Hydrolase, Protease, Serine protease	Overexpression of TMPRSS4 reduces E-cadherin and induces N-cadherin and vimentin in A549 lung cáncer cells, supporting an EMT phenotype. These changes are accompanied by enhanced migration, invasion and tumorigenicity in vivo. Analysis of 70 NSCLC samples from patients revealed a very significant correlation between TMPRSS4 expression and CSC markers ALDH ($p = 0.0018$) and OCT4 ($p = 0.0004$), suggesting that TMPRSS4 is associated with a CSC phenotype in patients' tumors [377]. MPRSS4 expression is associated with postoperative recurrence. In addition, the current survival curves demonstrated that TMPRSS4 expression is associated with statistically significant differences in survival among patients with lung adenocarcinoma [378]. The upregulation of TMPRSS4, partly ascribed to the downregulation of miR-125a-5p, promotes the growth of human lung adenocarcinoma via the NF-κB signaling pathway [379]. The TMPRSS4 knock down in H358, H441 and H2170 cells resulted in a significant reduction in proliferation, clonogenic capacity and invasion. A significant ($P<0.05$) decrease in the lung colonisation and growth was found when mice were injected with TMPRSS4-depleated H358-derived clones, as compared with controls. Expression of TMPRSS4 showed a >30-fold increase ($P<0.001$) in tumours in comparison with non-malignant samples. Levels in tumours with squamous cell carcinoma (SCC) histology were found to be significantly higher ($P<0.001$) than those with adenocarcinoma (AC) histology, which was confirmed in data retrieved from the microarrays. Kaplan–Meier curves demonstrated that high levels of TMPRSS4 were significantly associated ($P=0.017$) with reduced overall survival in the patients with SCC histology, whereas no correlation was found for the AC histology [380]. Knockdown of TMPRSS4 reduced the proliferation rate in several lung cáncer cell lines. When lung cáncer cell lines were treated with 5-aza-2′-deoxycytidine or trichostatin A, their proliferation rate and TMPRSS4 mRNA expression levels were also reduced through the upregulation of TFPI-2 by decreasing its methylation in vitro [381]. Transmembrane protease, serine 4 (TMPRSS4) is upregulated in IPF lungs and increases the fibrotic response in bleomycin-induced lung injury [382]. Abrogation of TMPRSS4 in H358 and H2170 cell lines caused a very strong reduction in proliferation (>70%, 96h after plating), clonocenicity (>90%, after 15 days in culture) and subcutaneous tumor growth. Reduction in S and G2/M phases of the cell cycle, increased apoptosis, and changes in gene expression of cell replication- and migration-promoting genes (i.e. MCM6, TYMS and CDKN1A(p21)) were also found. Cells lacking TMPRSS4 were highly sensitized to chemotherapy, including cisplatin, paclitaxel and gemcitabine, which significantly enhanced the antiproliferative, antitumor and proapoptotic effect of these drugs [383].
**TRPV2**	Calcium-permeable, non-selective cation channel with an outward rectification. Seems to be regulated, at least in part, by IGF-I, PDGF and neuropeptide head activator. May transduce physical stimuli in mast cells. Activated by temperatures higher than 52 degrees Celsius; is not activated by vanilloids and acidic pH. Calcium channel, Ion channel. Calcium transport, Ion transport, Transport. Ligand Calcium	TRPV2 transcripts have been detected in many resident macrophage populations, such as liver Kupffer cells, skin epidermal Langerhans cells and lung alveolar macrophages [384].
**UBE2T**	Accepts ubiquitin from the E1 complex and catalyzes its covalent attachment to other proteins. Catalyzes monoubiquitination. Involved in mitomycin-C (MMC)-induced DNA repair. Acts as a specific E2 ubiquitin-conjugating enzyme for the Fanconi anemia complex by associating with E3 ubiquitin-protein ligase FANCL and catalyzing monoubiquitination of FANCD2, a key step in the DNA damage pathway. Also mediates	Overexpression of UBE2T in NIH3T3 cells significantly promoted colony formation in soft agar medium ($p < 0.001$) [385]. In non-small cell lung adenocarcinomas, UBE2T and DTL were also amplified in around 7% of cases and linked with disease recurrence after surgical resection [386]. UBE2T silencing inhibited non-small cell lung cáncer cell proliferation and

	monoubiquitination of FANCL and FANCI. May contribute to ubiquitination and degradation of BRCA1. In vitro able to promote polyubiquitination using all 7 ubiquitin Lys residues, but may prefer 'Lys-11'-, 'Lys-27'-, 'Lys-48'- and 'Lys-63'-linked polyubiquitination. Transferase. DNA damage, DNA repair, Ubl conjugation pathway. ATP-binding, Núcleotide-binding.	invasion by suppressing the wnt/β-catenin signaling pathway [387].
**WFDC1**	Has growth inhibitory activity. Protease inhibitor, Serine protease inhibitor	WFDC1 expression was also dramatically downregulated in highly prolific mesenchymal cells and in various cáncers including fibrosarcomas and in tumors of the lung [388],
**ZEB1**	Acts as a transcriptional repressor. Inhibits interleukin-2 (IL-2) gene expression. Enhances or represses the promoter activity of the ATP1A1 gene depending on the quantity of cDNA and on the cell type. Represses E-cadherin promoter and induces an epithelial-mesenchymal transition (EMT) by recruiting SMARCA4/BRG1. Represses BCL6 transcription in the presence of the corepressor CTBP1. Positively regulates neuronal differentiation. Represses RCOR1 transcription activation during neurogenesis. Represses transcription by binding to the E box (5'-CANNTG-3'). *Promotes tumorigenicity by repressing stemness-inhibiting microRNAs.* ZEB1-AS1	Both TGF-β- and MYC-induced EMT required ZEB1, but engaged distinct TGF-β-dependent and vitamin D receptor-dependent (VDR-dependent) pathways, respectively. Functionally, we found that ZEB1 causally promotes malignant progression of HBECs and tumorigenicity, invasion, and metastases in non-small cell lung cáncer (NSCLC) lines. Mechanistically, ZEB1 expression in HBECs directly represses epithelial splicing regulatory protein 1 (ESRP1), leading to increased expression of a mesenchymal splice variant of CD44 and a more invasive phenotype. In addition, ZEB1 expression in early stage IB primary NSCLC correlated with tumor-node-metastasis stage. These findings indicate that ZEB1-induced EMT and associated molecular changes in ESRP1 and CD44 contribute to early pathogenesis and metastatic potential in established lung cáncer. Moreover, TGF-β and VDR signaling and CD44 splicing pathways associated with ZEB1 are potential EMT chemoprevention and therapeutic targets in NSCLC [389]. Despite its growth-inhibiting effect, EGFR inhibitor-induced ZEB1 strongly promotes EMT-dependent resistance to EGFR inhibitors partially through NOTCH1, suggesting a multifunctional role for NOTCH1 in EGFR-mutated cells [390]. The function of ZEB1 as a tumor suppressor appears to be oncogenic driver specific as ZEB1 expression inhibited both soft agar and xenograph growth in EGFR-mutant NSCLC cell lines, while it promoted soft agar and xenograph growth in KRAS-mutant NSCLC. However, this tumor suppressive role of ZEB1 seems to be independent of its ability to induce EMT, given that ZEB1 induced EMT in both KRAS- and EGFR mutant cell lines [391].
